# HiFi long-read RNA sequencing enhances clinical diagnostics in rare disorders

**DOI:** 10.1038/s41431-026-02042-9

**Published:** 2026-03-10

**Authors:** Carolina Jaramillo Oquendo, Federico Ferraro, Htoo A. Wai, Heather Ferrao, Herma van der Linde, Evita Karelioti, Liz Tseng, Harsharan Dhillon, Sam Holt, David J. Bunyan, Laura Donker Kaat, Marieke van Dooren, Jeff Zhou, Sarah Ennis, John W. Holloway, Tjakko J. van Ham, Diana Baralle

**Affiliations:** 1 Human Genetics and Genomic Medicine, Human Development and Health, Faculty of Medicine, University of Southampton, Southampton, UK; 2 Department of Clinical Genetics, Erasmus MC, University Medical Center Rotterdam, Rotterdam, the Netherlands; 3 Pacific Biosciences, Menlo Park, CA USA; 4 GenomeScan B.V, Leiden, the Netherlands; 5 Wessex Genomics Laboratory Service, Salisbury District Hospital, Salisbury, UK; 6 NHS Central & South Genomic Medicine Service Alliance, University Hospitals Birmingham NHS Foundation Trust, Birmingham, UK

**Keywords:** Genetics research, RNA splicing, Genetic testing, RNA sequencing, Next-generation sequencing

## Abstract

Splice-disrupting variants are estimated to account for one-third of disease-causing variants, yet many remain underrepresented in clinical databases due to limitations in detecting splicing changes beyond canonical splice sites. Short-read RNA sequencing (RNA-seq) has proved to be a valuable complement in clinical practice to address this gap, however, the added value of long-read RNA-seq is unclear. We evaluated the potential of PacBio long-read RNA-seq to detect pathogenic splicing events in rare disorders, comparing its performance to short-read RNA-seq. Participants from the UK (*n* = 23) and the Netherlands (*n* = 2) with suspected splice-altering variants underwent long-read RNA-seq following the Kinnex full-length RNA protocol. HiFi reads from the Revio instrument were processed using the Read Segmentation and Iso-Seq workflow and then classified and filtered using Pigeon. Detection of disease genes was comparable with short reads, with fibroblast capturing more transcripts overall. Novel isoforms accounted for ~14% of detected transcripts in both tissues, increasing following cycloheximide treatment in fibroblasts and decreasing following globin depletion in blood. Transcript abundance estimates showed strong concordance between short- and long-read platforms (Pearson *r* = 0.86 and 0.61 in blood and fibroblasts, respectively). LRS captured 21 confirmed known events, and revealed additional transcript-level effects in eight cases. This included intron retention, multiple exon skipping, leaky splicing, variant phasing, and isoform switching. These results demonstrate that long-read RNA-seq enhances detection and interpretation of clinically relevant splicing events, supporting its integration into diagnostic workflows for rare diseases.

## Introduction

A third of disease-causing variants are estimated to disrupt mRNA splicing [1, 2]. Splice-affecting variants are often missed and are under-ascertained in clinical variant databases, as these are not limited to canonical splice sites [3–5]. RNA testing is a complementary tool to DNA testing that provides functional evidence and identifies pathogenic events missed by traditional methods [6–14]. Within the UK or the Netherlands, some healthcare providers offer specialised RNA studies conducted via targeted reverse transcription PCR (RT-PCR) or RNA-sequencing (RNA-seq). RT-PCR is useful for genes with low expression (<1 transcripts per million [TPM]) and low-level splicing events [15]. However, RT-PCR is a bespoke test for each patient, and is inherently limited by gene annotation, amplicon lengths and prior assumptions on splicing abnormalities. In contrast, RNA-seq is independent of individual patients; it is agnostic to the abnormal transcript(s) and can aid in identifying a variety of events without a priori assumptions. Most RNA-seq studies rely on short-read (SR) RNA-seq, which, although it has some advantages over RT-PCR, cannot produce full-length transcripts, resolve complex regions, and identify certain types of aberrant splicing events such as long stretches of intron retention [16, 17].

As SR RNA-seq is integrated into clinical practice, it is essential to assess the potential benefits of long-read (LR) RNA-seq in this context. Full-length transcripts can improve the assessment of splicing and quantification of transcript abundance. Additionally, longer reads have sufficient genomic context to map challenging regions (high repetition, polymorphism, or low nucleotide diversity), therefore increasing coverage of genes that standard SR sequencing struggles to capture.

Long-read sequencing platforms, including Oxford Nanopore Technologies (ONT) and Pacific Biosciences (PacBio), have significantly improved read accuracy and scalability, although with lower throughput compared to short-read platforms [18]. PacBio’s Revio system claims 99.95% (Q33) read accuracy with read lengths of 15–20 kb, a yield 3-4x higher, and a 15x higher throughput than their previous Sequel Ile system. PacBio’s Kinnex kit, based on the MAS-seq method, concatenates smaller amplicons into larger fragment libraries for higher RNA sequencing throughput [19]. Similarly, ONT offers LR RNA-seq based on cDNA-converted transcripts and native RNA molecules, bypassing the need for reverse transcription or amplification, enabling the detection of full-length transcript isoforms, RNA modifications, and poly(A) tail length from the same molecule. Data storage requirements for LRs tend to be much higher than SRs for the same yield in gigabases. ONT generally requires greater raw data storage and offers lower read accuracy than PacBio at comparable yields.

Given limited published evidence demonstrating the ability of LR RNA-seq to identify pathogenic splicing events missed by short-read approaches [20–22], we investigated the utility of PacBio Kinnex RNA-seq (selected for its accuracy and reduced storage demands) to support clinical interpretation and characterise aberrant splicing in rare genetic disorders previously analysed by short-read RNA-seq.

## Materials and methods

### Patient cohort

Participants were enrolled into the University of Southampton’s Splicing and Disease study with appropriate ethical approval (REC 11/SC/0269, IRAS 49685, ERGO 23056). The sub-cohort used herein is comprised of 23 individuals with a suspected Mendelian disorder assessed by UK clinical genetics services in whom a candidate variant of uncertain significance (VUS) may have been identified through conventional DNA-based testing. SR RNA-seq results for six of these individuals were previously published [14]. Two participants examined at the Department of Clinical Genetics, Erasmus Medical Center (Rotterdam, Netherlands) for whom diagnostic RNA-seq was performed were also enroled (Institutional-review-board MEC-2012-387). SR RNA-seq results for one of these individuals were previously published [23]. Informed consent was obtained, and all individuals or their legal guardians provided written consent to share anonymised clinical and analysis data. Genotype and phenotype details are reported in Supplementary Table 1.

### Short-read RNA-seq and analysis

Blood RNA extraction and sequencing were previously reported [14]. SR RNA-seq was generated at NovoGene (Hong Kong) with ≥70 million reads. FASTQ files were aligned to the human genome reference (GRCh38) with GENCODE annotation v38 [24] using STAR aligner v2.6.1c [25]. Gene counts were generated using Salmon v1.6.0 in alignment-based mode with the –gcBias flag [26].

Fibroblast cell culturing, cycloheximide (CHX) treatment, RNA extraction and sequencing were previously reported [13]. CHX is an inhibitor of nonsense mediated decay (NMD) and was employed to prevent degradation of transcripts containing a premature stop (potentially) associated with pathogenic variants or to demonstrate loss-of-function effect at mRNA level for selected variants. SR RNA-seq was generated at GenomeScan, Leiden, the Netherlands, with ≥40 million reads generated per sample. Trimmed reads were aligned to GRCh38 with HISAT2 v2.2.1 [27]. Transcripts per million were extracted using Stringtie v2.2.3 and annotations from GENCODE v38. Splicing junctions were quantified using regtools v1.0.0 [28].

### LR sequencing and bioinformatics pipeline

Kinnex RNA-seq data extracted from blood were generated using a Revio system in two batches; each sequencing run lasted 24 h. The first batch of libraries was sequenced at PacBio Menlo Park, CA, while the second batch was sequenced at the PacBio EMEA headquarters in London.

**Fig. 1:**
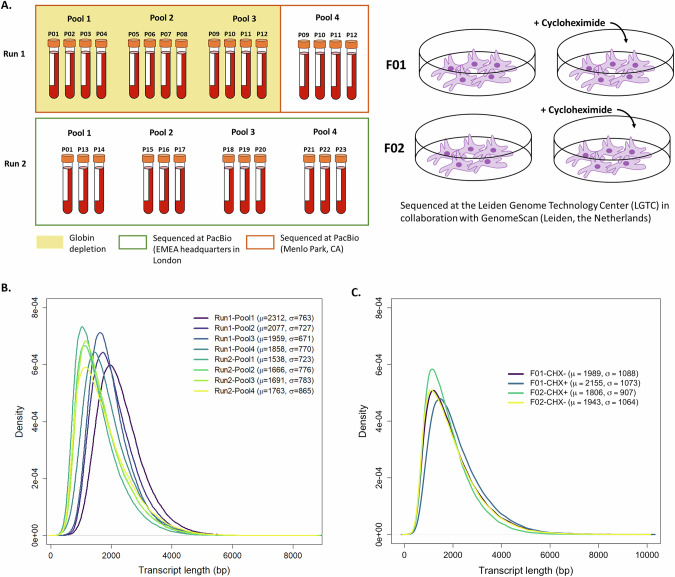
PacBio Kinnex transcriptome data quality assessment. **A** Sample preparation and sequencing overview. Left: Blood derived RNA samples were processed across two sequencing runs. Pools 1–3 in Run 1 underwent globin depletion, while Pool 4 in Run 1 and Pools 1–4 in Run 2 did not. Right: Fibroblast cell lines were treated with cycloheximide (+CHX) to stabilise transcripts subject to nonsense-mediated decay. **B**, **C** Distribution of read lengths across pools/SMRTcell in blood and fibroblasts, respectively. Mean and standard deviation are shown on the top right.

Run 1 was comprised of 16 libraries split across four pools (SMRTcells), sequencing 12 unique samples. Pools 1–3 consisted of four samples per SMRTcell and were globin-depleted. Pool 4 sequenced the same four samples in Pool 3 but with no globin depletion (Fig. 1A). Comparison of transcriptome profiles in Run 1 showed depletion reduced diversity, so the depletion step was omitted in Run 2 (Supplementary Fig. 1). To compensate for the reads that would be taken up by globin genes, the 12 libraries in Run 2 were split across four SMRTcells instead of three. Splicing and expression results for 22 of the 23 patients are reported. One patient was excluded due to lack of VUS.

HiFi reads were processed using the Read Segmentation and Iso-Seq workflow from SMRT Link v13.1. Kinnex arrays were segmented into their constituent cDNA reads using skera v1.2.0. Barcode removal and demultiplexing were performed with Lima v2.10.0. The Iso-Seq bioinformatics toolkit v4.1.2 was used to remove polyA tails, identify artefactual concatamers and cluster sequences, which were mapped against GRCh38_no_alt_analysis_set using pbmm2 1.14.0 in its specialised Iso-Seq mode. Remaining reads were collapsed using the PacBio Iso-Seq toolkit and then classified and filtered using Pigeon v1.2.0.

Fibroblast Kinnex RNA-seq data were generated using a Revio system at the Leiden Genome Technology Center (LGTC) in collaboration with GenomeScan (Leiden, the Netherlands), with sequencing runs of 24 h, and ≥10 million reads per sample. Two libraries were generated per individual, one library from fibroblasts treated with CHX+ and one CHX-, for a total of four libraries across one SMRTcell. Sequencing reads were processed with the Iso-Seq pipeline, specifically Iso-Seq v4.2.0, pbmm2 v1.16.0, and Pigeon v1.3.0, with the same reference and annotation used for the SR data.

RStudio v.4.4.2 was used for data visualisation and statistical analyses [29]. Enrichment analyses were also carried out in RStudio using EnrichR v3.4 [30].

### Assessment of aberrant splicing

To determine the functional consequence at a transcript level for each variant, both SR and LR RNA-seq data were loaded into the Integrative Genomics Viewer (IGV) [31], and each variant was examined as previously detailed [14]. Sashimi plots were generated with ggsashimi v.1.1.5 [32] following modification of the CIGAR strings in the aligned bams to ensure compatibility using an in-house script (https://github.com/f-ferraro/Kinnex-ggsashimi).

Aberrant splicing events from short- and long-read RNA-seq were collated into a comprehensive list and cross-checked to determine whether they were detected by the other platform. This approach allowed for a direct comparison of the sensitivity of each technology in identifying aberrant events.

## Results

### Quality assessment of Kinnex LR RNA-seq data

#### Blood

Sequencing depth varied across pools and runs, where Run 1 (*n* = 16 libraries; 4 per SMRTcell) had higher variability compared to Run 2 (*n* = 12 libraries; 3 per SMRTcell). Each library had ~13.2 million full length non-chimeric (FLNC) reads (range 4.9–24.3 million). Transcript length distributions had a consistent shape across runs and pools with slight shifts in peak positions. Notably, Run 1 pools tended to have higher mean transcript lengths compared to Run 2, with lower means and greater variability (Fig. 1B).

Globin depletion enhances detection of transcripts with lower expression in SR RNA-seq, as it reduces the representation of globin mRNA (~30% of transcripts), freeing up sequencing reads for more relevant transcripts [33–35]. To assess its utility on Kinnex data, we sequenced a pool of the same biological samples (*n* = 4) with and without globin depletion. Along with depletion of globin genes, genome-wide transcript diversity was negatively impacted by this procedure. Saturation curves of known genes and isoforms consistently showed higher number of detected genes and isoforms at the same depth for the undepleted libraries (Supplementary Fig. 1).

**Fig. 2:**
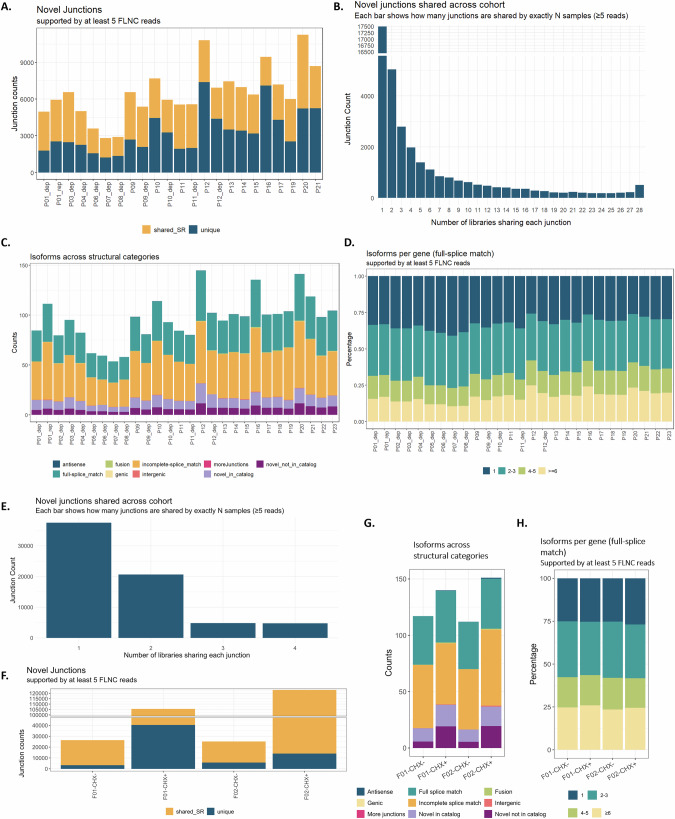
Characterisation of transcript diversity. **A** Fraction of novel junctions (supported by at least 5 FLNC reads) supported by paired short-read data in blood. Orange shows the number of junctions present in short reads (chr:start:end identical in both datasets). **B** Novel junctions shared across blood libraries. Each bar shows how many junctions are shared by exactly N libraries (supported by at least 5 FLNC reads). 17,846 junctions unique to a single library, and 508 junctions are shared across all 28. **C** Isoform distribution across structural categories in blood samples. **D** Isoform count per gene in blood libraries. Calculation includes only full-splice match isoforms and only those supported by at least 5 FLNC reads. **E** Novel junctions shared across fibroblast libraries. Each bar shows how many junctions are shared by exactly N libraries (supported by at least 5 FLNC reads). **F** Fraction of novel junctions (supported by at least 5 FLNC reads) supported by paired short-read data in fibroblasts. Orange shows the number of junctions present in short reads (chr:start:end identical in both datasets). **G** Distribution of isoforms across structural categories in CHX- and CHX+ fibroblasts. **H** Isoform count per gene in CHX- and CHX+ fibroblasts. Calculation includes only full-splice match isoforms and only those supported by at least 5 FLNC reads. CHX cycloheximide.

Iso-Seq is prone to overestimation of transcript diversity. To mitigate this, we performed Pigeon filtering to remove low-confidence isoforms, resulting in ~30% reduction of detected isoforms, in particular genic and intergenic sections of transcript (Supplementary Fig. 2A). We further validated junction support from LR-RNAseq using SR RNA-seq. On average 51% of novel junctions found within novel in-catalogue and not-in-catalogue transcripts (supported by ≥5 FLNC reads) were supported by the paired SR data (Fig. 2A). Additionally, 54% of novel junctions were present in at least two libraries, where 17,846 of novel junctions were unique to a single library and 508 were shared across all 28 (Fig. 2B).

#### Fibroblasts

Libraries from two patient samples were sequenced on a single SMRTcell (two libraries per sample: cycloheximide CHX+/CHX−) and obtained ~11.7 million FLNC reads per sample (range 11–12.6 million). Transcript-length distributions appeared uniform across the four samples (Fig. 1C), comparable to that observed for blood (Fig. 1B). Similarly, filtering of the detected isoforms by Pigeon resulted in the greatest reduction of intergenic, genic, and antisense categories (Supplementary Fig. 2B). Splice junction validation revealed that 45% of novel junctions (supported by ≥5 FLNC) were shared across at least two libraries (Fig. 2E). Paired SR RNA-seq libraries supported, on average, 82% and 75% of junctions in CHX- and CHX+ fibroblasts, respectively (Fig. 2F).

### High coverage and consistent quantification across short- and long-read RNA-seq

#### Blood

The Iso-Seq pipeline detected a minimum of 13,826 genes (31,132 isoforms) and a maximum of 18,642 genes (52,128 isoforms) annotated in GENCODE (Supplementary Fig. 3A, B) across the 28 libraries. To evaluate the diagnostic potential of LR RNA-seq, disease gene pick-up rate was also assessed, and at least 50% of both OMIM [36] and PanelApp [37] genes were detected across all libraries (Supplementary Fig. 3C, D). Transcript abundance estimates derived from SR and LR sequencing show strong concordance with a Pearson coefficient of 0.86 (Supplementary Fig. 3E), indicating high agreement across platforms.

#### Fibroblasts

In untreated fibroblasts, the Iso-Seq pipeline detected a minimum of 14,495 annotated genes, increasing to 15,994 in CHX-treated fibroblasts (Supplementary Fig. 4A, B). We observed that 64.6% of the genes detected in one untreated cell line were also detected in the other cell line, and this percentage increased to 69.4% in CHX+ cells. Biotype annotation of the 3541 genes detected only in either of the CHX+ fibroblast lines showed that these genes were mainly lncRNAs (36.1%), followed by protein coding (29.1%) and readthrough transcripts (24.4%) (Supplementary Fig. 4C). Further enrichment analysis in Enrichr, did not reveal a statistically significant enrichment in any of the sets included in the three Gene Ontology classes (cellular component, molecular function, biological process), Human phenotype ontology, nor OMIM.

Detected transcripts also increased after CHX treatment, likely related to transcripts normally undergoing nonsense mediated mRNA decay (Supplementary Fig. 4D, E). Importantly, the number of genes/transcripts discoverable appeared to be already saturated at the achieved read depth (Supplementary Fig. 4A, B, D, E). At equal sequencing depth, blood samples express, on average, a higher number of GENCODE-annotated genes and transcripts (Supplementary Fig. 5A, B). However, fibroblasts encompass a greater number of disease-relevant genes compared to blood [13] (Supplementary Fig. 4F). Correlation between level of expression estimated with short- and long-reads in fibroblasts was lower than what was observed in blood but still high with a Pearson coefficient of 0.646 and 0.669 in CHX+ and CHX- lines, respectively (Supplementary Fig. 4G).

### Assessment of identified transcripts

#### Blood

Using isoforms supported by ≥5 FLNC reads, the largest structural category annotated by Pigeon was incomplete-splice match (ISM), followed by full-splice match (FSM) isoforms. Among FSM isoforms, just over 30% of detected genes were represented by a single isoform and another 30% by 2–3 isoforms. Approximately 15% of genes showed high isoform diversity represented by six or more isoforms (Fig. 2C, D).

#### Fibroblasts

Most of the isoforms detected in the CHX- fibroblasts were annotated as FSM and ISM categories, similar to blood (Fig. 2G). CHX treatment led to a higher number of transcripts classified as novel not in catalogue, i.e. transcripts that use novel donors and/or acceptors not present in GENCODE annotation (Fig. 2G). Across the four cell lines, most genes had 2–3 distinct isoforms, while a similar proportion of genes were represented by a single or ≥6 isoforms (Fig. 2H).

### LR RNA-seq improves resolution and variant interpretation in select cases

Across SR and LR RNA-seq, 27 distinct splicing outcomes were identified across all patient samples. LR RNA-seq detected: 18 aberrant splicing events linked to a variant, three normal splicing outcomes, two splicing events supported by a low number of reads and failed to capture four outcomes (Table 1). Three of the four events missed by LR were due to low/no coverage of the gene (*PHF8, COX7B* and *KIAA0825*); *PHF8* was likely attributed to a poor sequencing run. The transcript abundance of *COX7B* decreased approximately 14-fold in blood and 5-fold in fibroblast from SR to LR sequencing, however, the underlying reason remains unclear. The remaining two events were not detected, likely due to their inherently low expression levels.

**Table 1:** Description of resulting aberrant splicing/expression events across patient cohort.

sample ID	Gene	Variant(s)	Resulting aberrant splicing events	SR RNA-seq	LR RNA-seq	Blood TPM (SR | LR)	Fibroblast TPM (SR | LR)	Length transcript (bp)
P01*	*UBR4*	NM_020765.3:c.8488+3 A > G	Intron 57 retention r.8488_8489ins8488 + 1_8489-1 p.(Ser2831ArgfsTer23)			15.6|75.9	55.4|131.2	15,892
P02	*KLHL7*	NM_001031710.3:c.936+3_936+22del	Exon 6-7 skipping, r.619_936del, p.(Val207_Lys312del) Note: Donor loss observed in LR, but intron retention cannot be confirmed. Normal transcript also detected by LR RNA-seq.	failed		5.6|11.4	24.5|43.2	5619
P03*	*NF1*	NM_000267.3:c.1168_1179del, p.(Asn390_His393del)	Exon 10 skipping r.1063_1185del, p.(Asp355_Lys395del)			6.0|7.8	27.3|76.7	12,381
P04*	*PTEN*	NM_000314.8:c.553 C > G, p.(His185Asp)	No aberrant splicing, r.=			46.8|58.9	43.9|22.5	8515
P05	*KLHL7*	NM_001031710.3:c.936+3_936+22del	Exon 6-7 skipping, r.619_936del, p.(Val207_Lys312del) Note: Donor loss observed in LR but intron retention cannot be confirmed.	failed	**□**	5.6|11.4	24.5|43.2	5619
P06	*NF2*	NM_000268.4:c.885+5 G > A	Exon 9 skipping r.1604_1792del, p.(Glu535_Lys597del)			3.4 | 3.7	57 | 82.7	5950
P07	*COX7B*	NM_001866.3:c.40+5 G > A	No aberrant splicing, r.=		**□**	4.4|0.3	30.2|7.7	2444
P08*	*RPS7*	NM_001011.4:c.507+3 A > G	Increased intron 6 retention, r.507_508ins507 + 1_508-1, p.(Val170GlyfsTer15)			70.8|20.7	521.6|198.2	732
P10	*PUF60*	NM_078480.3:c.560 T > A, p.(Leu187*)	No aberrant splicing, r.=			18.8|168.5	261.6|237.6	1868
P11*	*PHF8*	NM_015107.3:c.784-2 A > G	Exon 8 skipping r.784_946del, p.(Glu263GlyfsTer6)		**□**	7.5|3.2	26.9|11.0	6357
			Exon 8 and exon 7 skipping r.597_946del, p.(Leu200ValfsTer23)					
P12	*COL9A2*	NM_001852.4:c.1792+5 G > A	Exon 30 skipping r.1604_1792del, p.(Glu535_Lys597del)			1.2|4.1	1|0.3	2852
P13*	*PNKP*	NM_007254.4:c.1029+2 T > C	Exon 11 skipping, r.937_1029del, p.(Phe313_Pro343del)			12.0|19.1	54.6|22.1	1731
			Intron 10 and 11 retention, r.936_937ins936 + 1_937-1;r.1029_1030ins1029 + 1_1030-1	**□**				
P14	*WDR45B*	NM_019613.4:c.143-5 T > A	Exon 3 skipping, r.142_244del, p.(Glu48Ter)			9.9|21.2	232.4|142.3	2496
P15	*ITPR1*	NM_001378452.1:c.1712 A > G	Alternative donor exon 17, r.1713_1714ins1713 + 1_1713 + 17, p.(Glu572ValfsTer9) Note: LR RNA-seq allowed phasing with second variant NM_001378452.1:c.2659 C > T			35.0|16.5	2.8|1.3	354,159
P16	*KIAA0825*	NM_001145678.3:c.3451_3456+13del	Increased exon 18 skipping, r.3297_3456del, p.(Cys1099TrpfsTer4)		**□**	4.0|3.0	1.4|1.6	7241
P17	*EFTUD2*	NM_004247.4:c.1393 A > G, p.(Met465Val)	Alternative donor exon 15, r.1393_1411del, p.(Ser466AlafsTer3)			17.6|44.4	79.8|72	4326
P18	*ZMYM2*	NM_197968.4:c.3301+5 G > A	Exon 20 skipping, r.3133_3301del, p.(Gly1045LeufsTer4)	Not done		19.8|34.9	19.5|16.5	7429
P19	*SETD5*	NM_001080517.3:c.-177 + 1 G > A	No aberrant splicing, r.= Note: Donor loss observed in SR and LR but intron retention cannot be confirmed			8.5|30.0	57|49	6931
P20	*MLH1*	NM_000249.4:c.704 A > G, p.(Asp235Gly)	Exon 9 skipping, r.678_790del, p.(Glu227SerfsTer42)			8.5|15.7	837.5|1978.9	3178
			Increased exon 9 and 10 skipping, r.678_883del, p.(Glu227PhefsTer11)	**□**				
P21	*BAP1*	NM_004656.4:c.581 G > A, p.(Gly194Glu)	No aberrant splicing, r.=			7.6|25.8	83.0|14.5	3600
P22	*LMNA*	NM_170707.4:c.1381-5 G > A	Alternative acceptor intron 7, r.1380_1381ins1381-1_1381-5, p.(Asp461GlyfsTer21)			1.9|27.1	837.5|1978.9	3178
P23	*PTEN*	NM_000314.8:c.634+3 A > C	Increased exon 6 skipping; r.493_634del, p.(Gly165IlefsTer9)	**□**		46.8|58.9	43.9|22.5	8515
F01*	*TCOF1*	NM_001371623.1:c.2860-3215_2860-3214insN[3396]	No aberrant splicing, r.=; isoform switch with exonization of portion of the SVA			3.0|6.9	57|49	5026
F02	*YY1 – SLC25A29 & SLC25A47*	NM_152333.4:c.-120-994_*23708del	Read-through transcript			17.3|71.8	56.7|41.5	2247

*TPM* Median transcripts per million derived experimentally, *SR* short-reads, *LR* long-reads, *Failed* sent for short read RNA-seq but failed QC rendering data unusable,  Event detected,  Event detected but low confidence (i.e., <5 reads supporting event), **□** Event not detected. * in Sample ID column indicates samples for which RNA-seq or RT-PCR results have been previously published.

Where gene coverage was sufficient, LRS confirmed aberrant splicing found previously, and in eight cases (P01, P02, P09, P13, P15, P20, P23), LRS detected additional effects on transcripts, enhancing interpretation of variants and either helping resolve pathogenicity or providing additional biological insights into the variant’s effect. This included identification of intron retention events with more confidence (P01, P13), i.e. did not need to validate with RT-PCR or intronic reads not present in other samples, phasing variants of interest (P15, F01), and quantifying both known and novel transcripts (P02, P09, P20, P23, F01). Detailed figures/results for all samples with aberrant splicing are detailed in Supplemental Results, and we highlight a few examples in the next sections.

#### NM_001011.4(*RPS7*):c.507+3A > G skews expression toward unannotated intron retained transcript

**Fig. 3:**
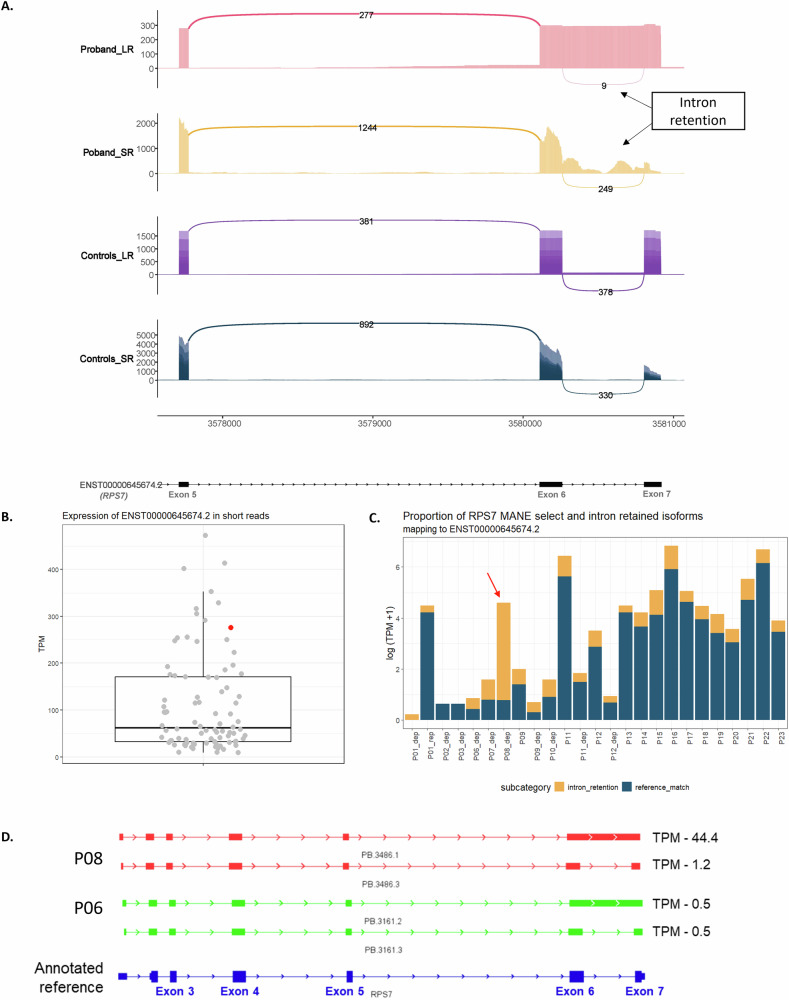
*RPS7* isoform characterisation. **A** Sashimi plot comparing splicing patterns between proband and controls. Both short-read (SR) and long-read (LR) data are shown. **B** TPM values of ENST00000645674.2 (*RPS7*) calculated by Salmon in short read data across 88 samples. Transcripts per million (TPM) value for proband highlighted in red. **C** Proportion of MANE select transcript (blue) vs intron 6 – retained (orange) transcript across blood libraries. Red arrow points to bar reflecting the proband. **D** Visualisation of transcripts identified in proband (red), P06 as control (green) and annotated reference (blue).

*RPS7* encodes a ribosomal protein essential for ribosome biogenesis and function; variants in this gene have been associated with Diamond-Blackfan anaemia [38, 39], consistent with the patient’s (P09) phenotype. SR sequencing suggested potential intron 6 retention; however, due to presence of intronic reads in controls results were inconclusive (Fig. 3A). Proband P09 had also previously undergone RT-PCR, which yielded normal results [15]. Salmon was used to quantify the transcript abundance of the MANE Select transcript (ENST00000645674.2/NM_001011.4) in SR RNA-seq. When compared to 87 unrelated samples, quantification of ENST00000645674.2/NM_001031710.3 in SRs suggested a slight increase in expression of this transcript (Fig. 3B). LR RNA-seq confirmed the variant did lead to intron 6 retention, however, intron 6 retention was also observed in controls, explaining RT-PCR results. Both patient and control samples exhibited two transcript isoforms: one retaining intron 6 and the MANE Select transcript (Fig. 3A, C, D). In controls, the intron-retained isoform was present at the same or lower levels than the MANE Select transcript. In the patient this intron retained transcript was ~40 times more abundant. This significant shift in transcript ratio could lead to RPS7 protein deficiency, likely due to competition of the aberrant isoform. While SRs were able to pick up the potential intron retention, the difference in isoform usage was only made clear with the long-read data.

#### Homozygous NM_001031710.3(*KLHL7*):c.936+3_936+22del variant causes leaky splicing

Biallelic *KLHL7* variants are known to cause PERCHING syndrome, a rare multisystemic developmental disorder [40, 41]. Previous RT-PCR assay identified exon skipping caused by NM_001031710.3:c.936+3_936+22del variant, leading to variant reclassification. LR RNA-seq confirmed these results but also identified a complete splice match to the MANE Select transcript (NM_001031710.3), see Supplemental Results Fig. 2. While a low-level event, no normal transcripts were expected as this is a homozygous variant, indicating leaky splicing. This patient has most of the constituent features of PERCHING syndrome, as previously described [41] (patient 6), not consistent with an attenuated phenotype. However, this patient is ambulant and still living at age 17 and perhaps not as profoundly disabled as some of the more severe cases.

#### LRS facilitates variant phasing

Proband P15 was initially referred for splicing assessment of variant NM_001378452.1:c.1712A > G in exon 17 of *ITPR1*. Both SR and LR RNA-seq showed the presence of an alternative donor site in intron 17, leading to an out-of-frame insertion (Supplemental Results – Fig. 10). After receiving the LR results, the referring diagnostic team contacted us to investigate phasing for a second variant in exon 23 (NM_001378452.1:c.2659C > T), previously unreported to us. LR RNA-seq successfully provided phasing information, revealing that the second variant was in trans with the initial variant. This was a complex clinical case where the clinical team ultimately reported the two variants as VUSs, mainly because there is currently insufficient evidence to support that null alleles are a mechanism of disease in autosomal recessive *ITPR1-*related disorders.

#### Retrotransposon-induced isoform switch in *TCOF1*

**Fig. 4:**
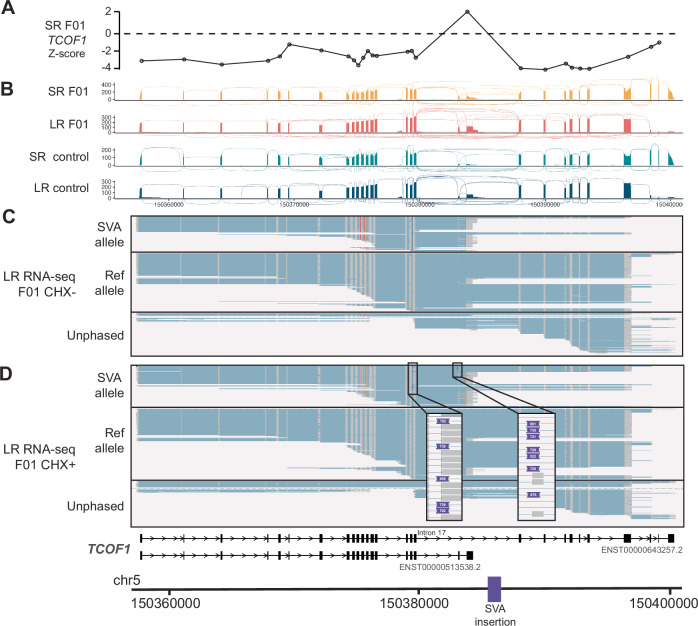
Detection of full abnormal transcripts due to *TCOF1* SVA insertion in F01 fibroblasts. **A** Z-score plot showing the relative expression level of the exons of *TCOF1* estimated from SR RNA-seq. **B** Sashimi plot showing level of expression of exons and splicing junctions observed in SR and LR RNA-seq data from F01 and an unrelated control. CHX+ and CHX- have been summed up. **C** Phased FLNC data from F01. The SVA-carrying allele shows preferential expression of the shorter *TCOF1* isoform compared to the reference allele. Inset show exonized SVA insertion in the longer transcripts. **D** Schematic of genomic coordinates and transcripts in the locus, and of the SVA insertion.

Loss-of-function variants in *TCOF1* are the most common cause of Treacher-Collins syndrome [42], and recently, a retrotransposon insertion as novel pathogenic mechanism in this gene was reported [23]. SR RNA-seq outlier-analysis suggested an isoform switch from the canonical *TCOF1* transcript (ENST00000643257.2/NM_001371623.1) to a shorter transcript (ENST00000513538.2, Fig. 4A, B), lacking a nucleolar localisation signal expected to impair ribosome biosynthesis [43]. SR RNA-seq suggested skewing of one allele (allele frequency 0.33, based on four heterozygous SNVs), partially restored by CHX (allele frequency 0.39). However, no pathogenic event was identified. Characterization by ONT LR genome sequencing and direct RNA-seq [23] showed insertion of a SINE-VNTR-Alu (SVA) retrotransposon into *TCOF1* intron 17. The SVA is partially exonized, inducing an early termination codon leading to nonsense-mediated mRNA decay.

We performed Kinnex LR RNA-seq (Fig. 4B–D) in fibroblasts from patient F01, carrying this pathogenic event. Based on full-splice and incomplete-splice match transcripts, *TCOF1* expression was TPM 12.7 in CHX- and TPM 8.3 in CHX+ (compared to TPM 13.4 and 6.36 in CHX- and CHX+ fibroblasts from F02, respectively). Manual inspection of the FLNC of CHX- fibroblasts from F01 and phasing of the reads by heterozygous SNVs, showed the isoform switch induced by the SVA: while the reference allele produced mostly long *TCOF1* isoforms (TPM 8.7 long isoform vs 0.9 short isoform, similarly to what was observed in the two haplotypes in F02 average TPM 2.7 vs 0.5), the allele carrying the SVA produced mostly shorter *TCOF1* isoforms (TPM 1 vs 2.1) (Fig. 4C). This was confirmed by the data from the CHX+ fibroblast RNA (TPM 6 vs 0.24; SVA-allele TPM 1.1 vs 0.9), where partial exonization of the SVA in 12 of the 16 reads supporting a long isoform were also detected (Fig. 4D). Notably, no SVA-containing isoform was reconstructed by the *Iso-Seq* pipeline, although the event was clearly evident in the raw sequencing data. While limitations in the bioinformatic analysis of LR RNA-seq still exist, the raw data completely resolved and phased full-length transcripts that included the SVA insertion that went undetected using SR RNA-seq.

#### Deletion of YY1 distal exons induces transcript readthrough

Loss-of-function *YY1* variants cause autosomal dominant Gabriele-de Vries syndrome [44]. SR RNA-seq identified *YY1* as an expression outlier in individual F02, presenting with phenotype consistent with the Gabriele-de Vries syndrome. Manual inspection of the SR RNA-seq data indicated read-through transcripts involving the locus of two genes downstream of *YY1*, i.e., *SLC25A29* and *SLC25A47* (Supplemental Results Fig. 18A, B). Reanalysis of exome data identified a heterozygous deletion of the last 3 exons of *YY1 (NM_00340*3*.5)* and exon 2 and 3 of the neighbouring gene *SLC25A29* (*NM_001039355.3*) (Supplemental Results Fig. 18C). LR RNA-seq (CHX+ and CHX- fibroblasts) from this patient, confirmed the presence of *YY1* transcripts including portions of one of the *SLC* genes or both (Supplemental Results Fig. 18B). LR data also confirmed partial intron retention around the annotated exons *SLC25A29* included in the read-through transcripts. Iso-Seq analysis showed reduced *YY1* expression in CHX-untreated fibroblasts from F02 (22.2 TPM) compared to F01 (51.7 TPM). It also reconstructed a *YY1* read-through into the *SLC25A29* locus in both samples but did not detect isoforms involving *SLC25A47*, as these occurred in fewer than two FLNC reads.

## Discussion

We applied PacBio full-length Kinnex RNA-seq in blood (±globin depletion) and fibroblasts (±cycloheximide) to assess throughput, compare its performance to SR technology, and evaluate its utility for clinical interpretation of variants referred for RNA-seq studies. Kinnex RNA-seq confirmed previous SR RNA-seq findings in blood and fibroblast samples. This new technology enhanced characterisation and facilitated interpretation of events that were only partially detectable by SR RNA-seq. In some cases, reanalysis of the SR RNA-seq using insights gained from LR sequencing can also improve interpretation.

### Limitations of LR RNA-seq

While our study demonstrates the potential of PacBio Kinnex LR RNA-seq for variant interpretation, several limitations remain. Despite a strong correlation between short- and long-reads (Pearson coefficients of 0.86 in blood; 0.66 in fibroblasts), coverage was lower for a subset of genes. In blood, pathway analysis revealed that these genes were predominantly immune-related, including T-cell receptor variable and immunoglobulin genes. For example, *PHF8* and *COX7B* had TPM values of 7.5 and 4.4, respectively, in SR data, compared to 3.2 and 0.3 in blood LR data, impairing variant assessment. In contrast, fibroblasts provided better resolution for these genes, with median TPMs of 30 and 27 in SR data, and 8 and 11 in LR data, respectively.

Interestingly, discrepancies in the predicted expression values were observed across both tissues. These may be attributed to differences in read depth, suggesting deeper sequencing (achievable with longer sequencing times) might be required to fully capture transcript diversity. Additionally, some transcripts may be subject to nonsense-mediated decay, further reducing their detectability and requiring more sensitive approaches, as might have been the case with the *SETD5* canonical splice site variant (P19).

Current bioinformatics pipelines for quantifying aberrant transcripts and identifying novel events are not yet optimised for LR RNA-seq [45]. Given the abundance of novel transcripts detected, distinguishing disease-relevant events from those arising from transcript diversity yet to be characterised or artefacts remains a challenge. We demonstrate that stricter filtering strategies (e.g. FLNC support) on top of specialised tools (e.g. Pigeon) can mitigate this issue. While half of novel junctions were supported by SR RNA-seq, the remaining junctions may still represent biologically relevant events, particularly in well-expressed genes where short-read mapping is difficult. Future work integrating proteomic data will be essential to assess which isoforms are translated and potentially functional. Additionally, validation across tissues could help distinguish ubiquitous transcripts from tissue-specific or disease-relevant splicing events. These complementary approaches will be key to realising the full diagnostic potential of LR RNA-seq.

### Technical considerations

Although our assessment of globin depletion and cycloheximide treatment was based on eight and four libraries, respectively, our data offer valuable insights into optimising experimental design. These include selecting appropriate library prep, tissue types, and determining the number of samples per SMRTcell to best adapt to the needs of the study. Strikingly, while globin depletion in blood is associated with higher transcript diversity in SR RNA-seq [33, 46], the opposite was observed with the Kinnex protocol. Variability in coverage was evident in the first run, and although we cannot attribute the variability to the depletion process, the non-depleted pools performed better. In fibroblasts, cycloheximide treatment enhances transcript and gene diversity using Kinnex, in line with observations in SR.

### Enhanced transcript resolution with LR RNA-seq

LR RNA-seq successfully identified additional events and provided a clearer understanding of aberrant splicing and isoform usage. Compared to SR RNA-seq, LR RNA-seq can better detect intron retention events with less noise and events that span multiple exons. Moreover, it enables full-length transcript detection, quantification of transcript diversity, and allele-specific expression quantification.

At least 10% of detected transcripts were novel, not in the catalogue, and this could have significant implications when standard annotations are used to quantify gene expression. This was exemplified with the quantification of *RPS7* by Salmon in proband P09, where no annotated transcripts include the retention of intron 6. This demonstrated how relying solely on SR RNA-seq quantification of specific isoforms can be misleading. There is large variability in the quantification of this transcript using SR RNA-seq, and only with the LR RNA-seq is the differential isoform usage apparent.

LR RNA-seq also revealed leaky splicing in *KLHL7*. While not altering clinical interpretation of the variant, leaky splicing could explain variability across disorders caused by variants in the same gene. Similarly, LR data for the *MLH1* variant (Supplemental Results – Fig. 15) also detected additional exon 9–10 skipping at similar ratios to the exon 9 skipping initially detected in SR data. These observations underscore the added value of LR RNA-seq in uncovering subtle splicing events that may be contributing to disease heterogeneity [47, 48].

Furthermore, having full-length transcripts, or at least very long reads, enables transcript phasing as evidenced in probands P15 and F01. Therefore, broadening access to testing for patients where parental DNA/RNA is unavailable, where we can still assess *cis*/*trans* expression of variants, if variants are not spliced out.

### Clinical integration and future directions

LR RNA-seq is a powerful and increasingly accessible tool for detecting aberrant splicing and gene expression in rare disease diagnostics. Recent advances have significantly improved throughput, though costs remain higher than SR sequencing, which also benefits from well-established pipelines. For cases with a variant of uncertain significance in a well-expressed gene (TPM ≥ 5) and outside repetitive regions, SR RNA-seq remains a practical first-line test. However, LR RNA-seq provides critical transcript-level resolution and can refine or validate SR findings. Alternatively, targeted deep long-read sequencing may also be applied to specific genes with a known VUS [49], though throughput is a consideration.

In the UK, SR RNA-seq is only just being introduced into clinical practice, so it will take time before LR RNA-seq becomes part of the NHS diagnostic framework. Similarly, in the Netherlands, diagnostic SR RNA-seq is available in only a few clinical centres. The next step is to develop LR methods for identifying relevant events in patients without a VUS, already being explored with SR RNA-seq [6–8, 13, 14], helping to determine which patients and samples would benefit most from this technology.

While LR RNA-seq has clear potential in rare disease diagnostics, its integration into clinical workflows is actively progressing and will benefit from continued optimisation of analytical pipelines, cost reduction and standardisation.

## Supplementary information

Supplemental Table 1

Supplemental Figures

Supplemental Results

## Data Availability

The datasets generated during and/or analysed during the current study are available from the corresponding author on reasonable request.

## Code availability

We made use of publicly available tools listed in the methods with default parameters. We recommend using the latest update of these tools.
