# Supplementary material for: HiFi long-read RNA sequencing enhances clinical diagnostics in rare disorders
Source: Eur J Hum Genet. 2026 Mar 10;34(6):840–51. doi: 10.1038/s41431-026-02042-9 (PMC13247170; doi:10.1038/s41431-026-02042-9)
Supplement: Supplementary file 1 — Supplemental Table 1 [file 41431_2026_2042_MOESM1_ESM.docx]

**Supplementary Table 1.** Description of genetic variants, genotypes (**GT**) and observed phenotypes. **AL**: acceptor loss, **DL**: donor loss, **AG**: acceptor gain, **DG**: donor gain. * Indicates samples for which short read RNA-seq or RT-PCR results have been previously published.

| ID | Gene | Variant(s) | Location | GT | Phenotype | Tissue | SpliceAI Δ score AL\|DL\|AG\|DG |
| --- | --- | --- | --- | --- | --- | --- | --- |
| P01* | *UBR4* | NM_020765.3:c.8488+3A>G | Intron 57 | HET | Cerebellar ataxia, nystagmus | Blood | 0.04\|0.26\|0.03\|0.13 |
| P02 | *KLHL7* | NM_001031710.3:c.936+3_936+22del | Intron 7 | HOM | Perching syndrome | Blood | 0.68\|0.97\|0.0\|0.01 |
| P03* | *NF1* | NM_000267.3:c.1168_1179del, p.(Asn390_His393del) exon 10 | Exon 10 | HET | Neurofibromatosis type 1 | Blood | 0.03\|0.04\|0.0\|0.0 |
| P04* | *PTEN* | NM_000314.8:c.553C>G, p.(His185Asp) | Exon 6 | HET | Callouses palms and soles, prominent bleeding gums, macrocephaly. | Blood | 0.0\|0.0\|0.0\|0.0 |
| P05 | *KLHL7* | NM_001031710.3:c.936+3_936+22del | Intron 7 | HET | Unaffected carrier | Blood | 0.68\|0.97\|0.0\|0.01 |
| P06 | *NF2* | NM_000268.4:c.885+5G>A | Intron 9 | HET | Intra-medullary ependymoma. Sibling with ependymoma | Blood | 0.53\|0.52\|0.0\|0.0 |
| P07 | *COX7B* | NM_001866.3:c.40+5G>A | Intron 1 | HET | Pupil asymmetry, cerebellar hypoplasia, Ligamentous laxity, cataplexy, Learning difficulties, Microcephaly, right foot neuropathy. | Blood | 0.0\|0.02\|0.01\|0.01 |
| P08* | *RPS7* | NM_001011.4:c.507+3A>G | Intron 6 | HET | Diamond Blackfan Syndrome | Blood | 0.0\|0.03\|0.0\|0.08 |
| P10 | *PUF60* | NM_078480.3:c.560T>A, p.(Leu187*) | Exon 7 | HET | PUF60-related developmental disorder | Blood | 0.0\|0.01\|0.01\|0.01 |
| P11* | *PHF8* | NM_015107.3:c.784-2A>G | Intron 7 | HEMI | Global developmental delay, epilepsy and hypotonia | Blood | 0.99\|0.59\|0.27\|0.0 |
| P12 | *COL9A2* | NM_001852.4:c.1792+5G>A | Intron 31 | HET | Stickler syndrome | Blood | 0.20\|0.75\|0.0\|0.37 |
| P13* | *PNKP* | NM_007254.4:c.1029+2T>C | Intron 11 | HET | Global developmental delay | Blood | 0.69\|0.93\|0.03\|0.06 |
| P14 | *WDR45B* | NM_019613.4:c.143-5T>A | Intron 2 | HOM | Structural brain abnormality and profound developmental delay | Blood | 0.82\|0.75\|0.0\|0.0 |
| P15 | *ITPR1* | NM_001378452.1:c.1712A>G | Exon 17 | HET | Ataxic cerebral palsy, global developmental delay | Blood | 0.01\|0.08\|0.0\|0.55 |
| P16 | *KIAA0825* | NM_001145678.3:c.3451_3456+13del NM_001145678.3:c.2020T>A, p.(Tyr674Asn) | Exon 18 / Intron 18,  Exon 12 | HET | Post axial polydactyly left hand and both feet. Normal development. Mild ear dysplasia and dysmorphism | Blood | 0.53\|0.80\|0.0\|0.09,  0.0\|0.0\|0.03\|0.03 |
| P17 | *EFTUD2* | NM_004247.4:c.1393A>G, p.(Met465Val) | Exon 15 | HET | Left Kidney Agenesis, Klippelfeil, scoliosis, arachnoid cyst | Blood | 0.0\|0.06\|0.0\|0.99 |
| P18 | *ZMYM2* | NM_197968.4:c.3301+5G>A | Intron 23 | HET | low muscle tone, developmental delay, problems with fine motor skills, squint. Low set ears | Blood | 0.66\|0.98\|0.01\|0.02 |
| P19 | *SETD5* | NM_001080517.3:c.-177+1G>A | Intron 1 | HET | Talipes, Hemihypertrophy, short stature, Speech delay. | Blood | 0.0\|0.98\|0.0\|0.16 |
| P20 | *MLH1* | NM_000249.4:c.704A>G, p.(Asp235Gly) | Exon 9 | HET | Transverse colon cancer | Blood | 0.29\|0.29\|00\|0.0 |
| P21 | *BAP1* | NM_004656.4:c.581G>A, p.(Gly194Glu) | Exon 8 | HET | BAP1-inactivated melanocytic tumour | Blood | 0.0\|0.0\|0.26\|0.0 |
| P22 | *LMNA* | NM_170707.4:c.1381-5G>A | Intron 9 | HET | dilated cardiomyopathy | Blood | 0.01\|0.01\|0.97\|0.0 |
| P23 | *PTEN* | NM_000314.8:c.634+3A>C | Intron 6 | HET | Macrocephaly, developmental delay, oropharynx-haematoma | Blood | 0.92\|0.98\|0.0\|0.0 |
| F01* | *TCOF1* | NM_001371623.1:c.2860-3215_2860-3214insN[3396] | Intron 17 | HET | Treacher Collins syndrome | Fibroblast | NA |
| F02 | *YY1 - SLC25A29* | NM_152333.4:c.-120-994_*23708del | Intron 2 – Intron 2 | HET | Gabriele-de Vries syndrome | Fibroblast | NA |
