# Supplementary material for: HiFi long-read RNA sequencing enhances clinical diagnostics in rare disorders
Source: Eur J Hum Genet. 2026 Mar 10;34(6):840–51. doi: 10.1038/s41431-026-02042-9 (PMC13247170; doi:10.1038/s41431-026-02042-9)
Supplement: Supplementary file 2 — Supplemental Figures [file 41431_2026_2042_MOESM2_ESM.docx]

**Supplementary Figures**

**
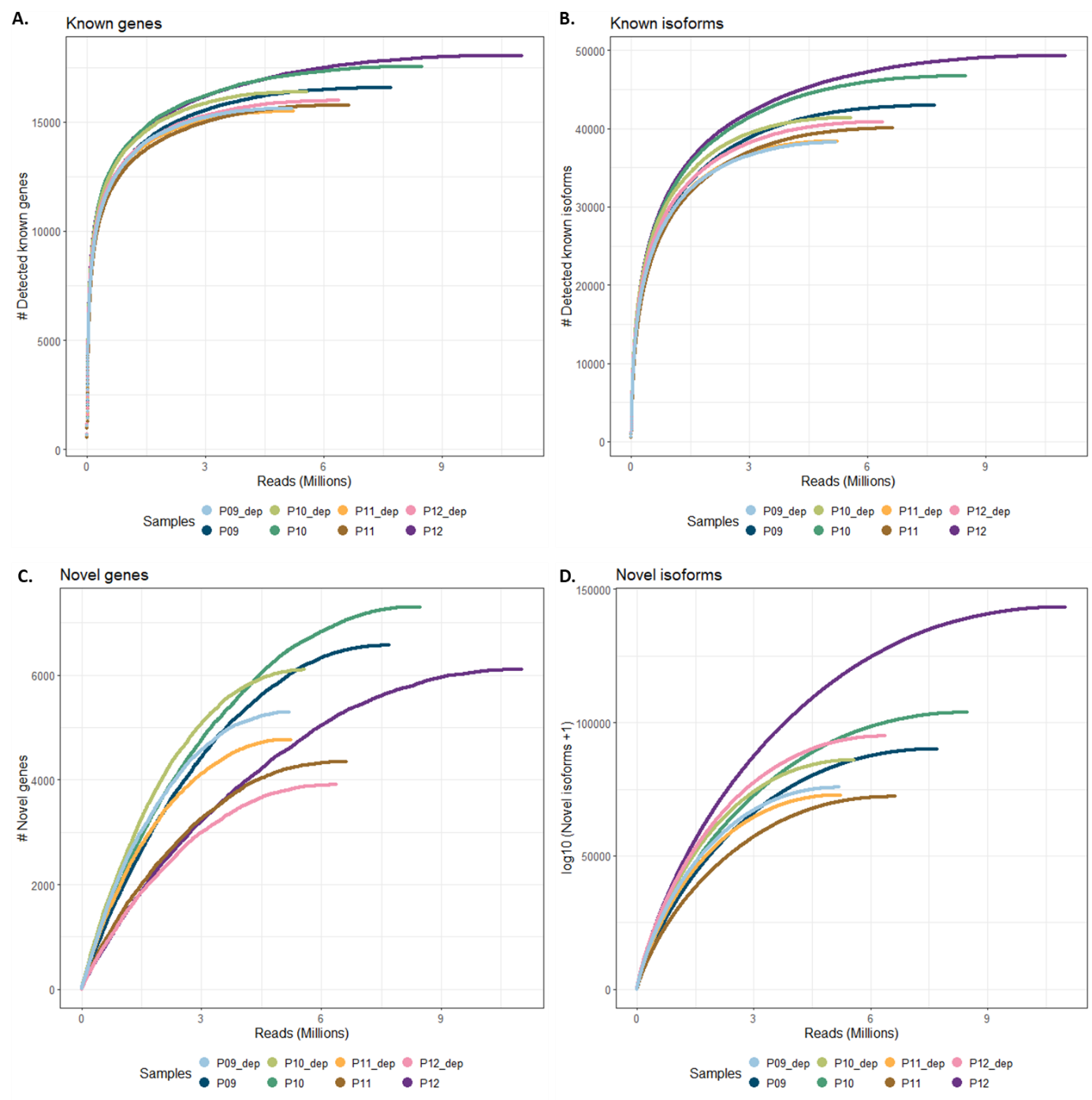
**

**Supplementary Figure 1**. Saturation curves comparing globin depleted vs undepleted libraries. **A-B.** Number of known genes and isoforms (present in GENCODE) detected by Iso-Seq pigeon in Run 1 Pools 3 and 4, across various levels of down sampled sequencing reads. **C-D.** Number of novel genes and isoforms (not present in GENCODE) detected by Iso-Seq pigeon in Run 1 Pools 3 and 4, across various levels of down sampled sequencing reads. Run 1 Pool 3 underwent globin depletion, while Run 1 Pool 4 did not. Libraries were sequenced from biological replicates, with “_dep” indicating globin depleted libraries.


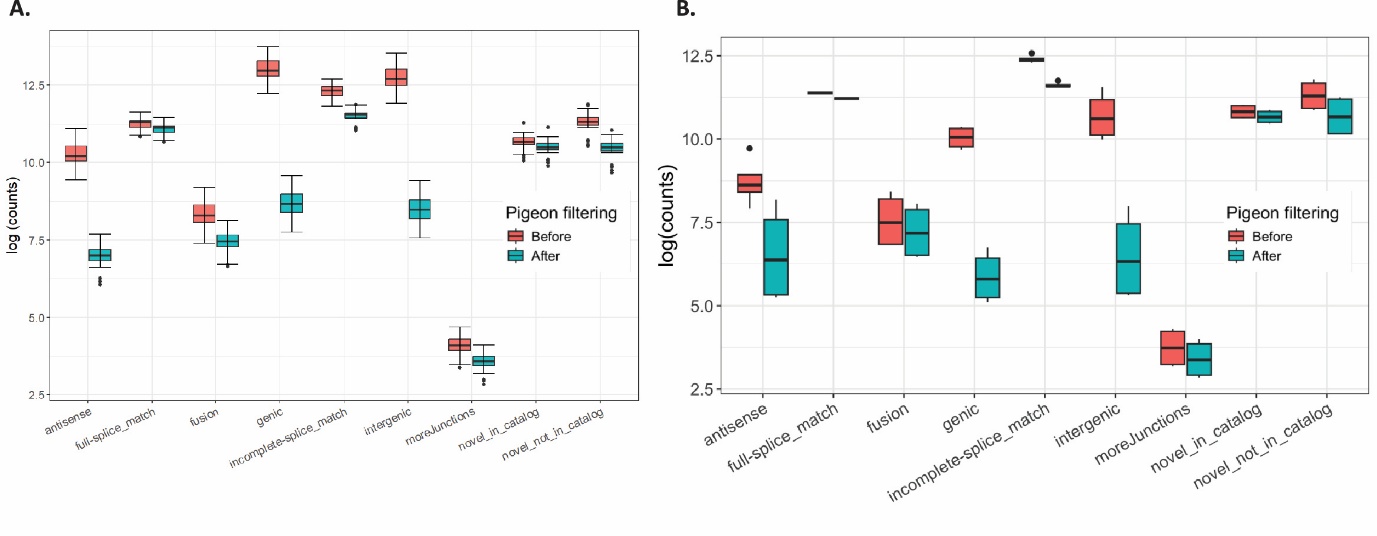
**Supplementary Figure 2.** Comparison of isoform count in each category before and after Pigeon filtering in **A.** blood and **B.** fibroblasts.


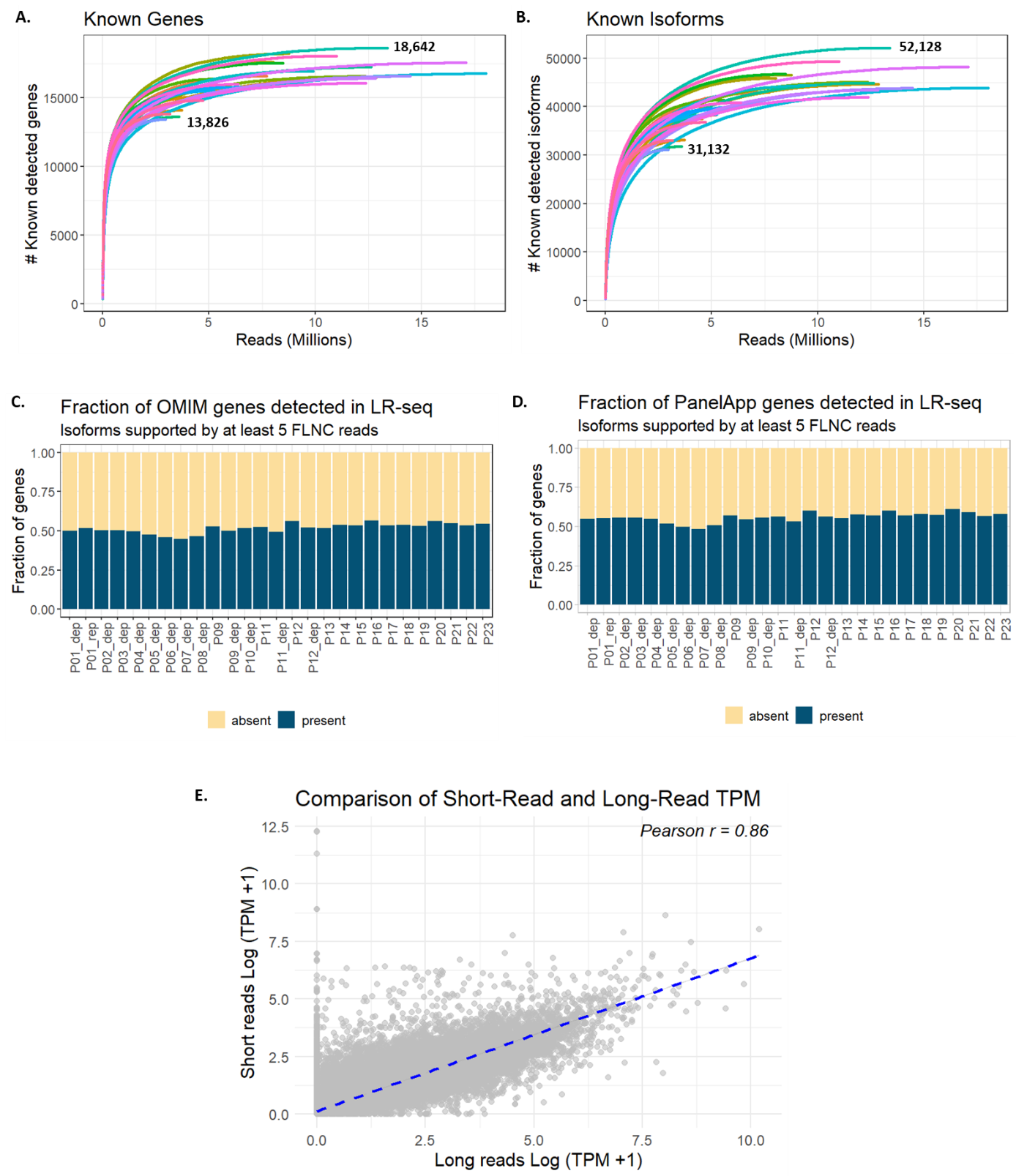


**Supplementary Figure 3.** Gene and isoform detection rates in blood. **A-B** Saturation plots for all blood libraries (n=28) showing the number of known genes and isoforms (present in GENCODE) across varying depths. The numbers show the min and max number of genes and isoforms detected. **C-D.** Fraction of OMIM (n=16,630) and PanelApp (n=3,643) genes detected by the Iso-Seq pipeline across all libraries. **E.** Correlation of transcript abundance measured in transcripts per million (TPM) in long versus short read RNA-seq.


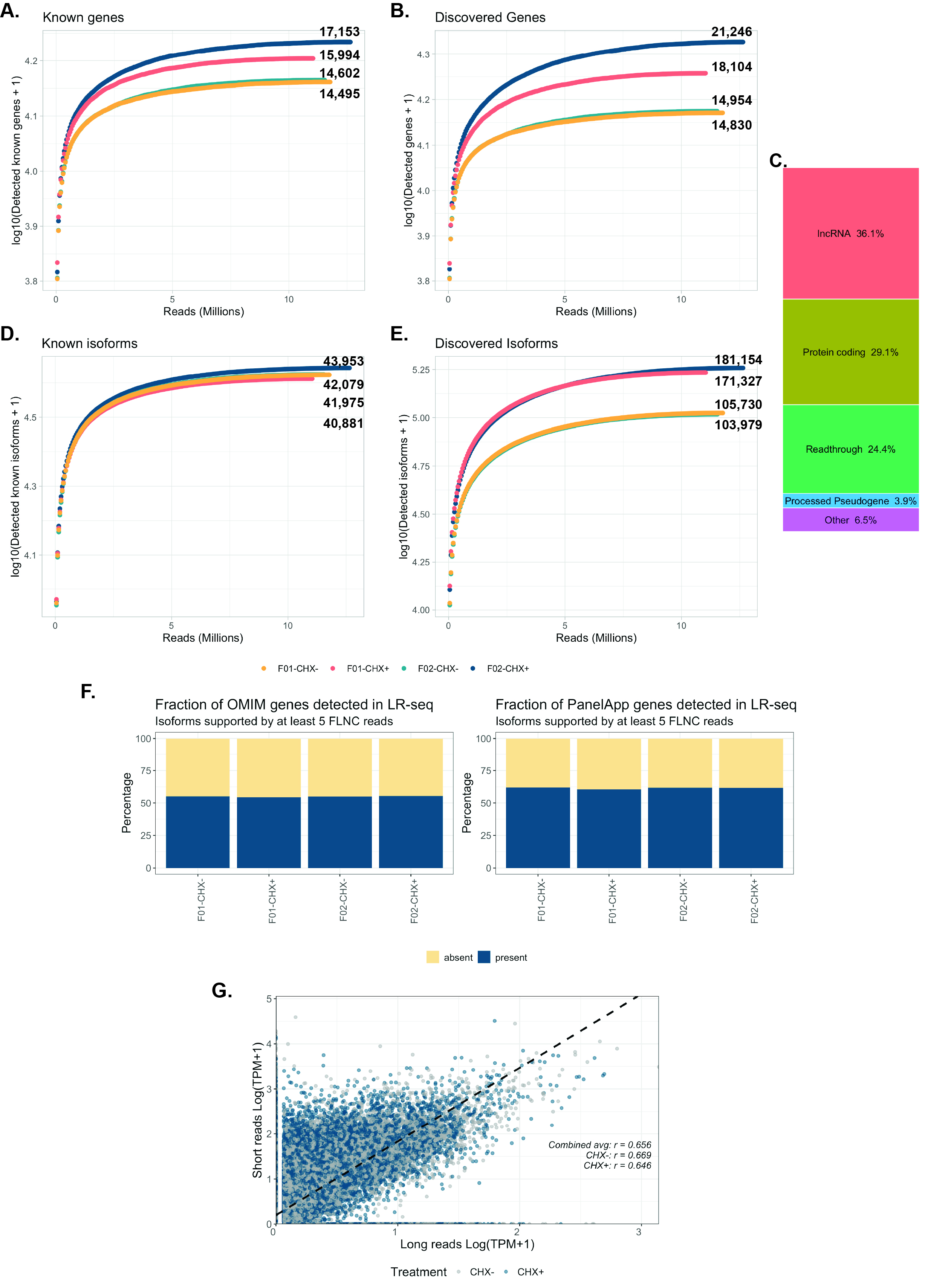


**Supplementary Figure 4.** Gene and isoform detection rates in fibroblast cell lines. **A-B.** Saturation curves showing the number of detected genes present in GENCODE or discovered by Iso-Seq pigeon from the fibroblast cell lines at different numbers of down sampled sequencing reads. Numbers indicate maximum detection achieved. **C.** Biotype annotation of genes detected only in cycloheximide-treated (CHX+) fibroblast cell lines. F01, F02 represent the two fibroblast lines; CHX- specifies lines not treated with cycloheximide, while CHX+ indicates cycloheximide-treated lines. **D-E.** Saturation curves for transcripts annotated in GENCODE or discovered from sequencing data by Iso-Seq pigeon at different numbers of down sampled sequencing reads. Numbers indicate maximum detection achieved. **F.** Fraction of OMIM (n=16,630) and PanelApp (n=3,643) genes identified by the Iso-Seq pipeline in the fibroblast lines. **G.** Correlation of transcript abundance measured in transcripts per million (TPM) in long versus short read RNA-seq from fibroblast lines.


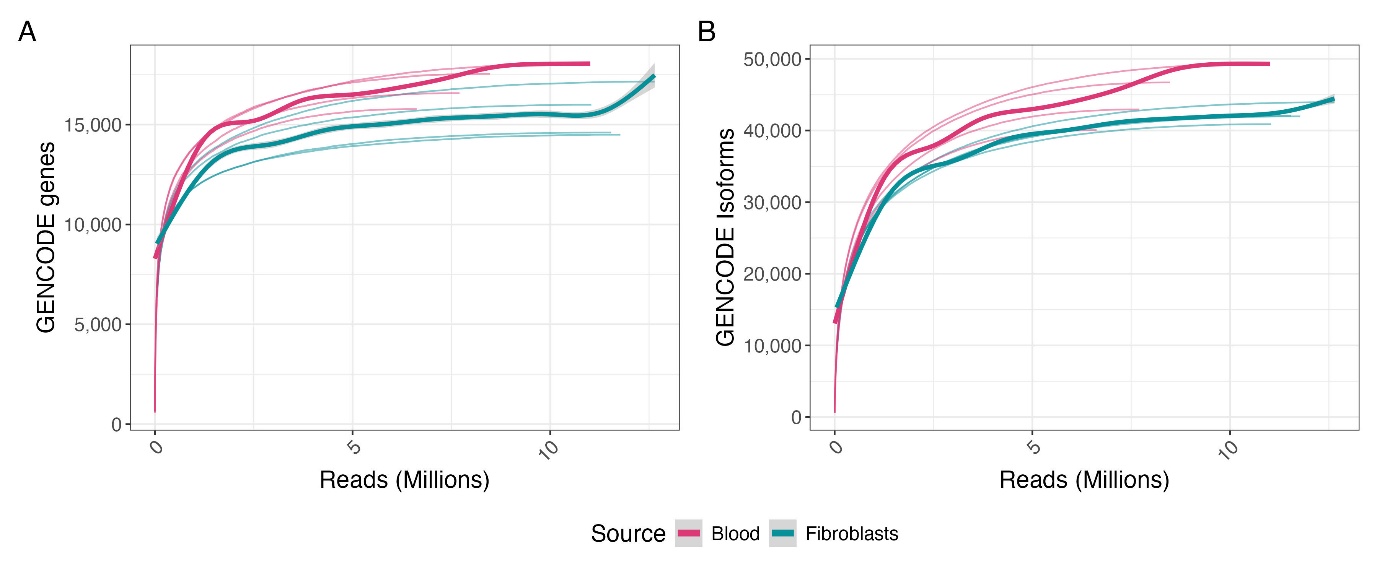


**Supplementary Figure 5.** Gene and transcript detection saturation analysis in blood and fibroblasts. Saturation curves showing the number of GENCODE-annotated **A** genes and **B** transcripts detected as a function of sequenced reads (in millions). Each thin line represents a patient sample, and the thick smoother curves represent the average. For blood, 4 random samples were used, for fibroblasts all 4 lines were used.
