# Supplementary material for: HiFi long-read RNA sequencing enhances clinical diagnostics in rare disorders
Source: Eur J Hum Genet. 2026 Mar 10;34(6):840–51. doi: 10.1038/s41431-026-02042-9 (PMC13247170; doi:10.1038/s41431-026-02042-9)
Supplement: Supplementary file 3 — Supplemental Results [file 41431_2026_2042_MOESM3_ESM.docx]

**Supplemental Results**

**P01 - NM_020765.3(*UBR4*):c.8488+3A>G [Heterozygous]:** Short reads showed an increase of intronic reads associated to the variant compared to controls (**Figure 1A**), indicating likely intron retention leading to an out-of-frame insertion r.8488_8489ins8488+1_8489-1 p.(Ser2831ArgfsTer23). However, due intronic reads within controls this event required validation. RT-qPCR also suggested increased intron retention, but the difference was modest (LinRegPCR efficiency for intron 57 was 1.852 and exon 44-45 (control assay) was 1.58. Long reads confirmed the intron retention event as seen in **Figure 1B**.
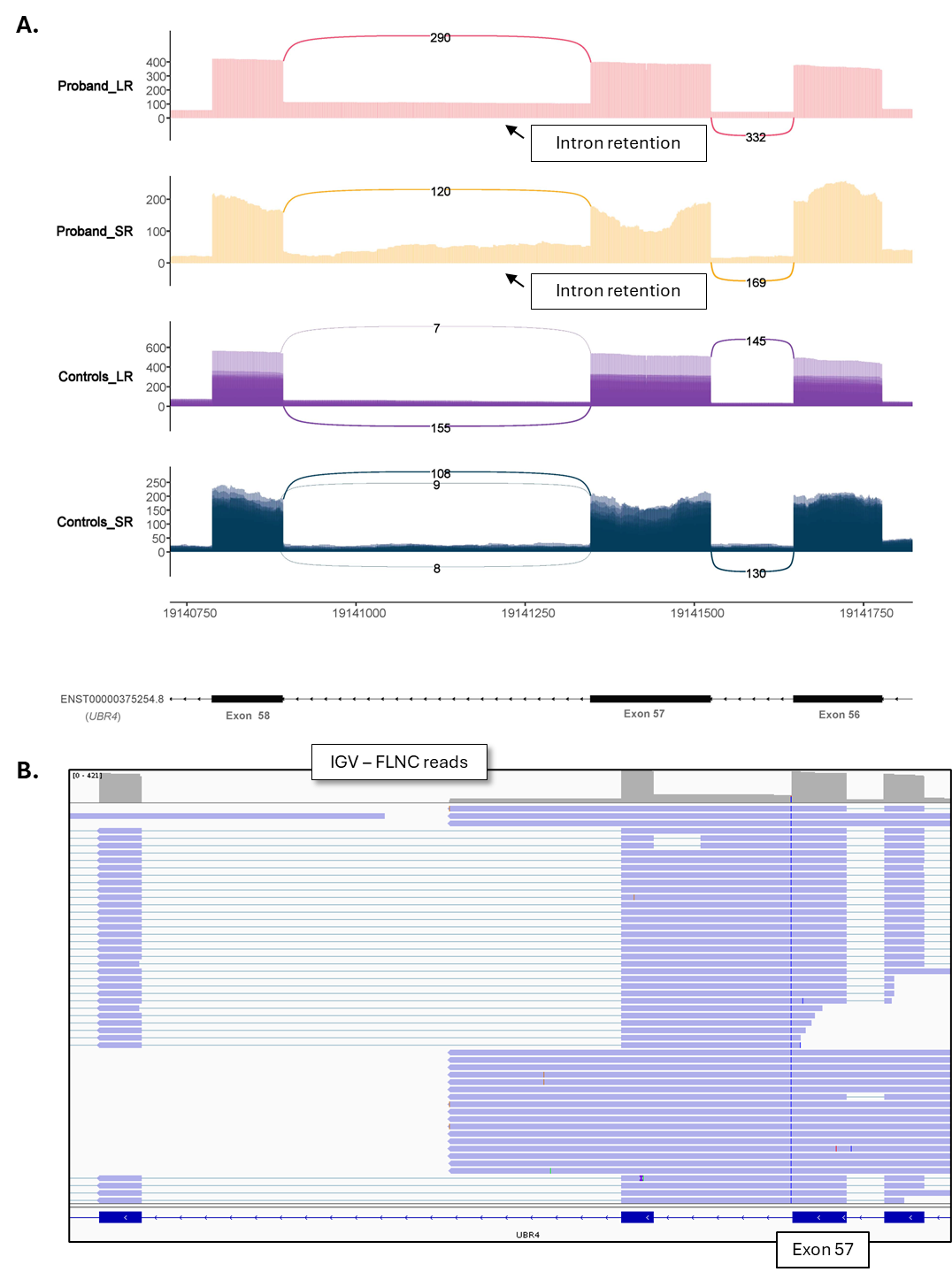


**Figure 1**. Transcript visualisation in proband P01. **A.** Sashimi plots comparing splicing patterns between probands and controls. Both short-read (SR) and long-read (LR) data are shown. **B.** Integrated Genomics Viewer (IGV) screenshot of full-length non-chimeric (FLNC) reads from proband. Long read data is presented without clustering

**P02 - NM_001031710.3(*KLHL7*):c.936+3_936+22del [homozygous]:** Short read RNA-seq failed for this sample, but previous RT-PCR result had shown exon skipping, which was subsequently used to reclassify the variant and offer carrier testing to family members. Long reads identified exon 6-7 skipping but also identified a full-splice match transcript. While low level, no normal transcripts were expected as this was a homozygous variant, suggesting leaky splicing (**Figure 2A**). Additionally, a potential intron retention event is indicated by reads that bypass the canonical donor splice site, showing the deletion and continued readthrough into the intronic region (**Figure 2B**).**
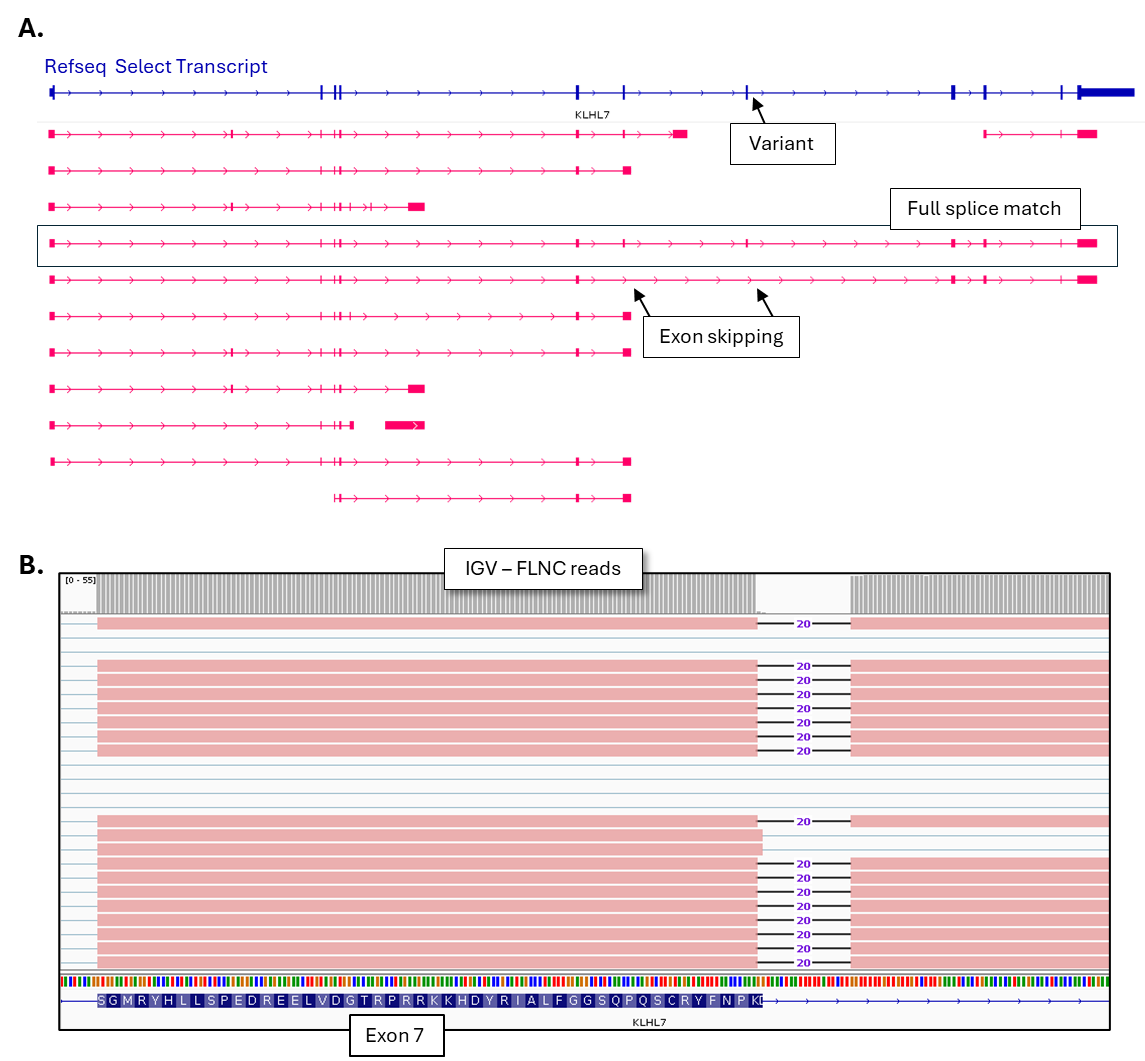
**

**Figure 2.** Transcript visualisation in proband P02. **A.** Schematic representation of KLHL7 transcripts aligned to the RefSeq Select Transcript (blue). Pink bars represent isoforms identified using Iso-Seq pipeline (post clustering and pigeon filtering) in proband P02. Exons are shown as thick boxes, and introns as connecting lines. Arrows indicate the direction of transcription. **B.** Integrated Genomics Viewer (IGV) screenshot of full-length non-chimeric (FLNC) reads from proband. Long read data is presented without clustering.

**P03 - NM_000267.3(*NF1*):c.1168_1179del, p.(Asn390_His393del) [Heterozygous]:** Short- and long-read sequencing detected exon 10 skipping (r.1063_1185del, p.(Asp355_Lys395del) in proband P03, absent from controls (**Figure 3A**). The deletion falls within a splicing regulatory motif, likely disrupting normal splicing recognition (**Figure 3C**). No additional events were detected with the LRS.
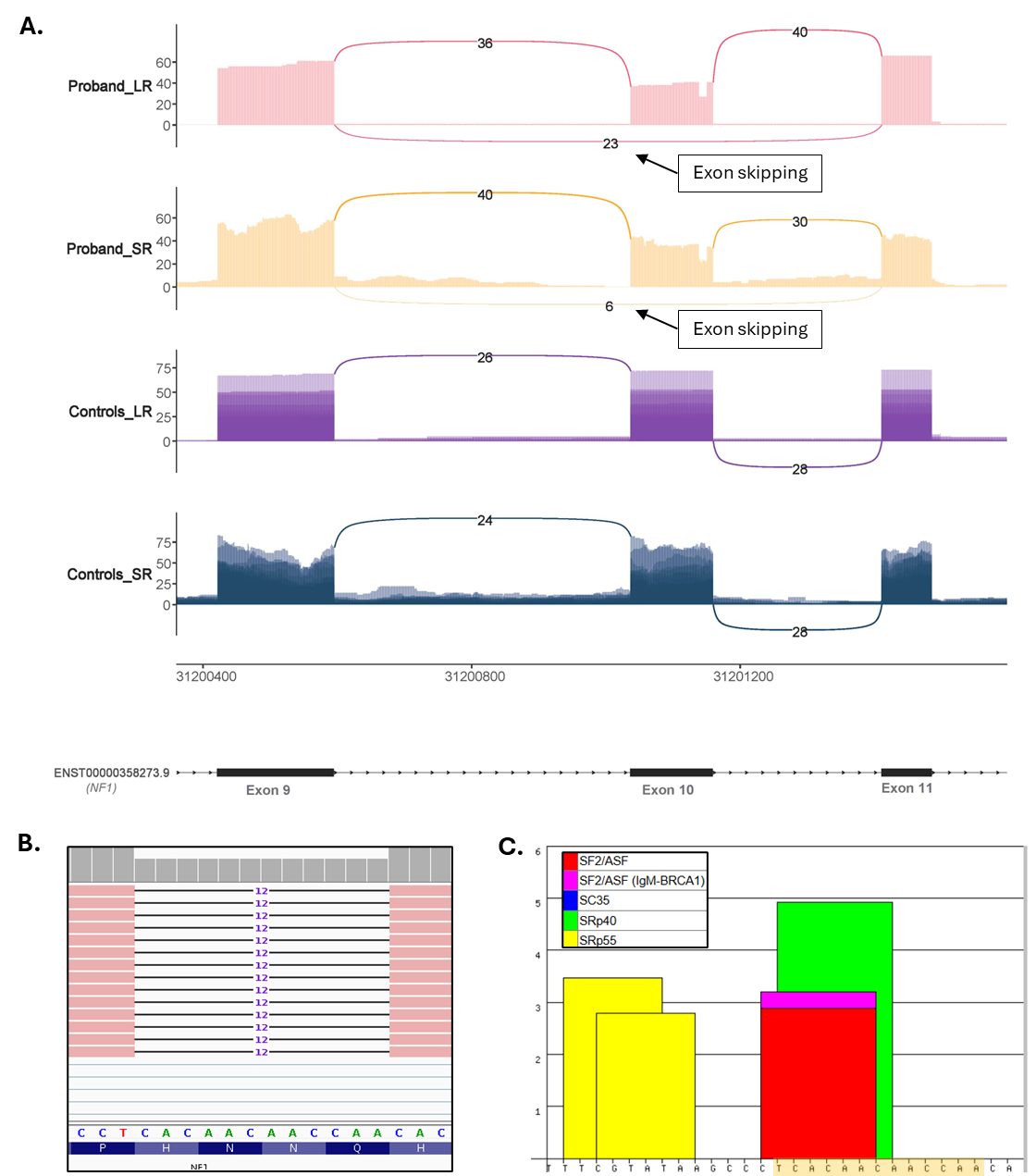


**Figure 3.** Transcript Visualisation in proband P03. **A.** Sashimi plots comparing splicing patterns between probands and controls. Both short-read (SR) and long-read (LR) data are shown. **B.** Integrated Genomics Viewer (IGV) screenshot of full-length non-chimeric (FLNC) reads showing the deleted sequenced. **C.** ESEfinder analysis showing that the deleted sequence (highlighted in yellow) overlaps predicted SF2/ASF and SRp40 binding motifs. This disruption likely impairs splicing enhancer function, providing a mechanistic explanation for the observed exon 10 skipping.

**P05 - NM_001031710.3(*KLH7*):c.936+3_936+22del [Heterozygous]:** This sample belongs to the unaffected parent of P02 for which short read RNA-seq also failed. Long-read sequencing did not detect any exon skipping (**Figure 4A**). However, we do observe reads showing the variant that bypasses the canonical donor splice site, showing the deletion and continued readthrough into the intronic region (**Figure 4B**).

**
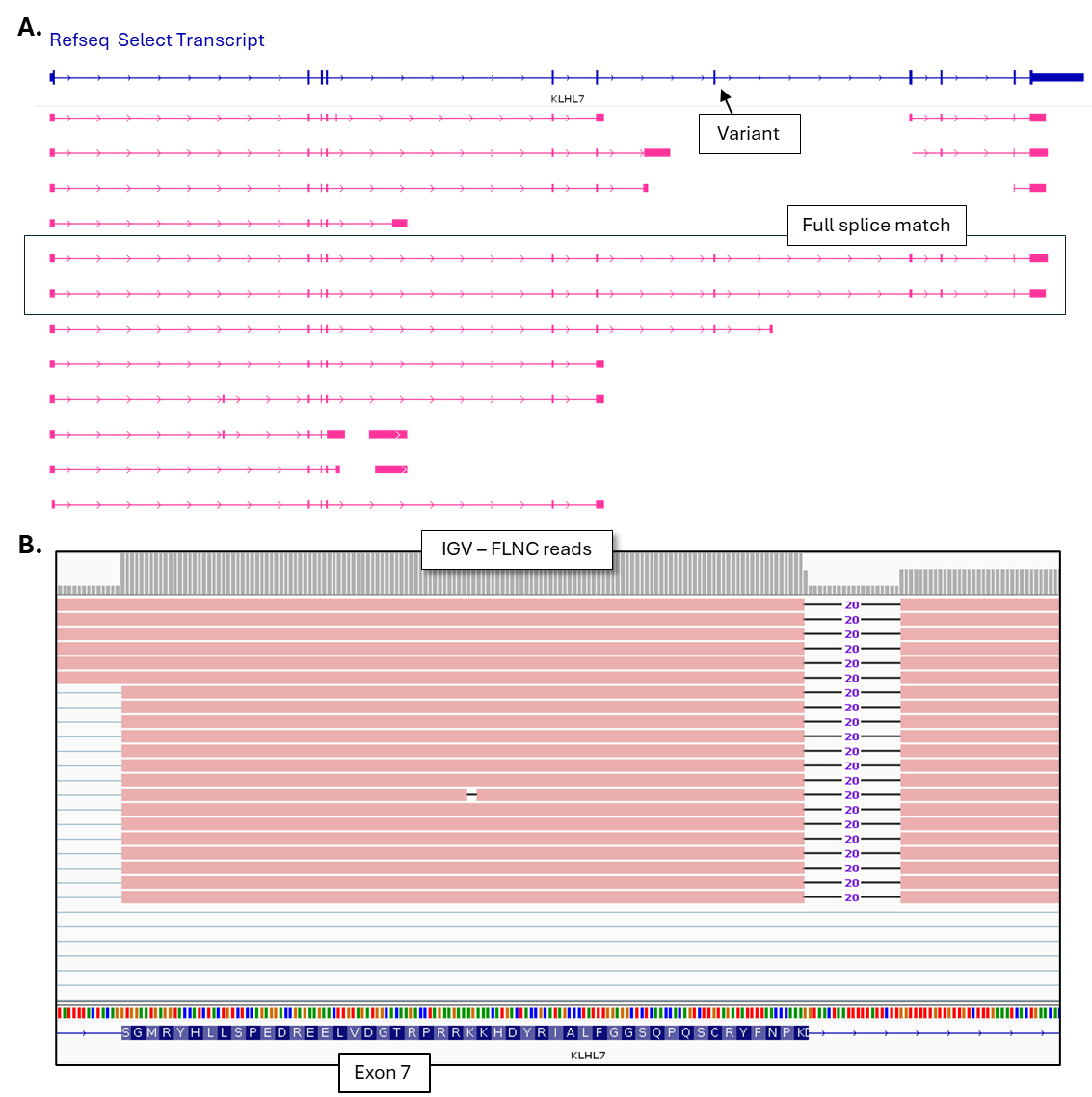
**

**Figure 4.** Transcript visualisation in unaffected parent of P02. **A.** Schematic representation of KLHL7 transcripts aligned to the RefSeq Select Transcript (blue). Pink bars represent isoforms identified using IsoSeq pipeline (post clustering and pigeon filtering) in proband P05. Exons are shown as thick boxes, and introns as connecting lines. Arrows indicate the direction of transcription. **B.** Integrated Genomics Viewer (IGV) screenshot of full-length non-chimeric (FLNC) reads from proband. Long read data is presented without clustering

**P06 - NM_000268.4(*NF2*):c.885+5G>A [Heterozygous]:** Short and long-read sequencing detected exon 9 skipping, resulting in r.1604_1792del p.(Glu535_Lys597del), absent from controls (**Figure 5A**). No additional events were detected with LRS.

**
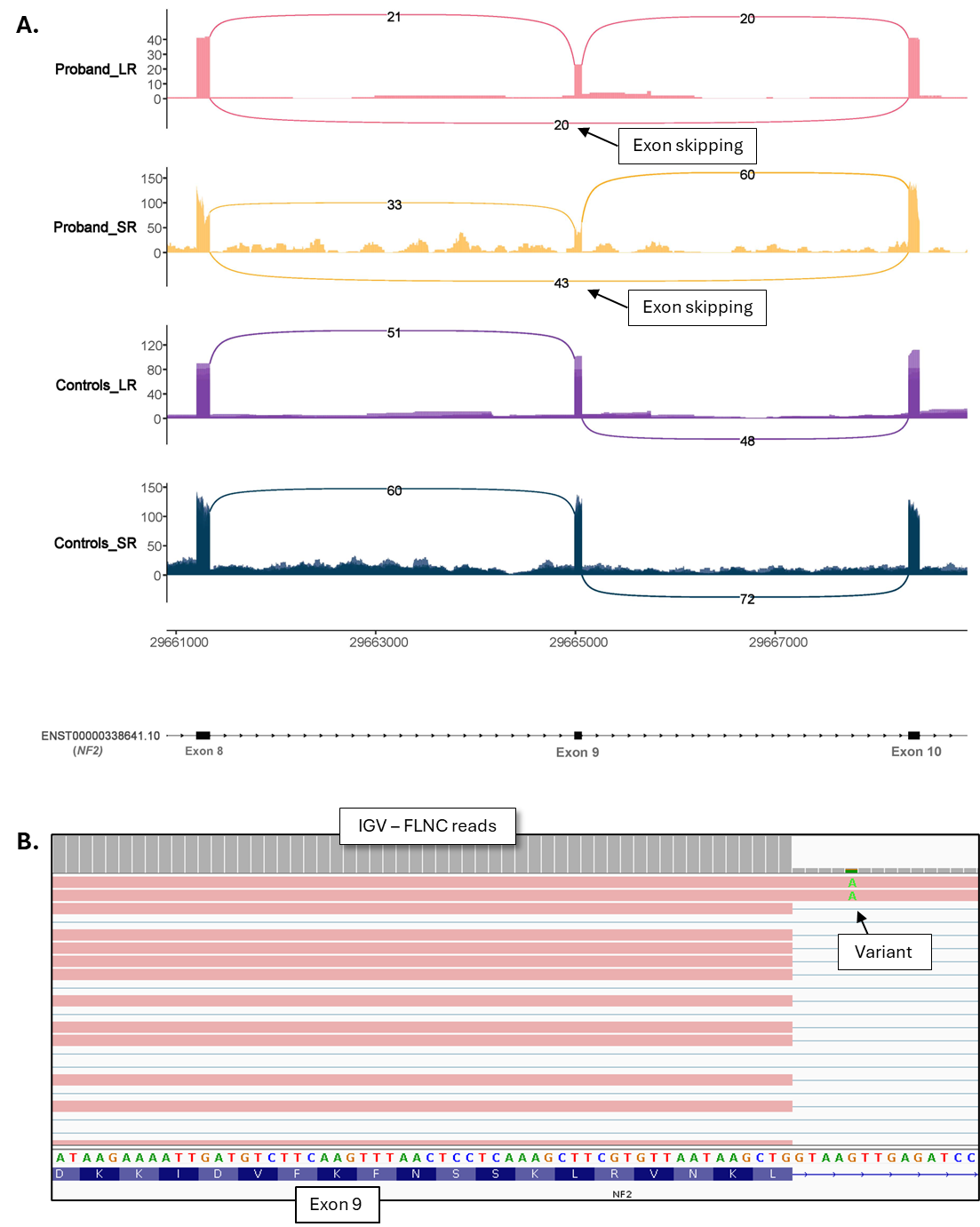
**

**Figure 5.** Transcript visualisation in proband P06. **A.** Sashimi plots comparing splicing patterns between probands and controls. Both short-read (SR) and long-read (LR) data are shown. **B.** Integrated Genomics Viewer (IGV) screenshot of full-length non-chimeric (FLNC) reads from proband. Long read data is presented without clustering.

**P11 - NM_015107.3(*PHF8*):c.784-2A>G [Hemizygous]:** Short read-sequencing identified two exon skipping events, skipping of exon 8 and exon 7-8. Long-read sequencing data (un-clustered aligned reads) did show evidence of exon 7-8 skipping, however the read depth for this gene was low and therefore the event was not detected after clustering and Pigeon filtering. **
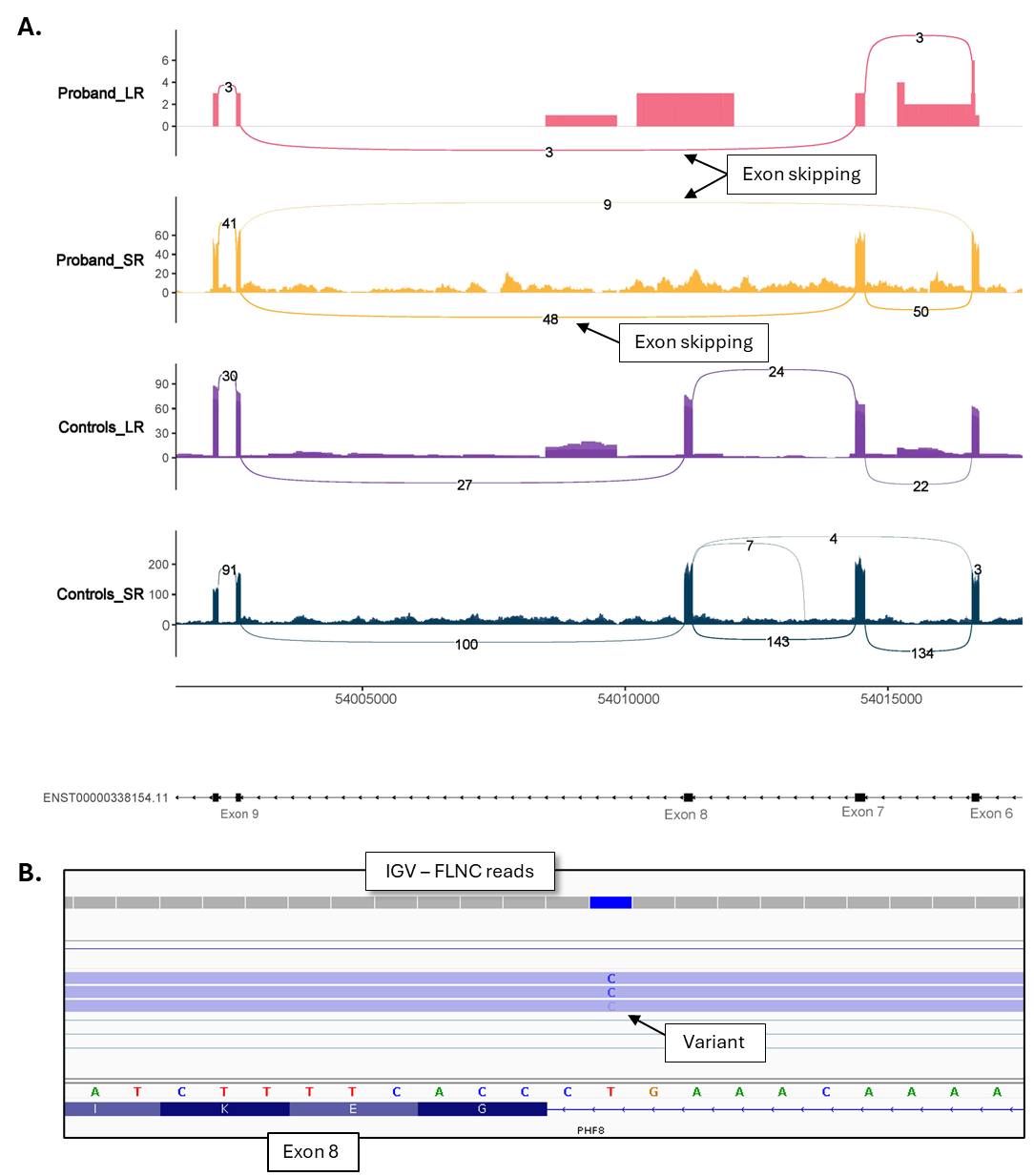
**

**Figure 6**. Transcript visualisation in proband P11. **A.** Sashimi plots comparing splicing patterns between probands and controls. Both short-read (SR) and long-read (LR) data are shown. **B.** Integrated Genomics Viewer (IGV) screenshot of full-length non-chimeric (FLNC) reads from proband. Long read data is presented without clustering.

**P12 - NM_001852.4(*COL9A2*):c.1792+5G>A [Heterozygous]:** Short and long-read sequencing detected exon 30 skipping resulting in r.1604_1792del, p.(Glu535_Lys597del), absent from controls (**Figure 7A**). No additional events were detected with LRS.**
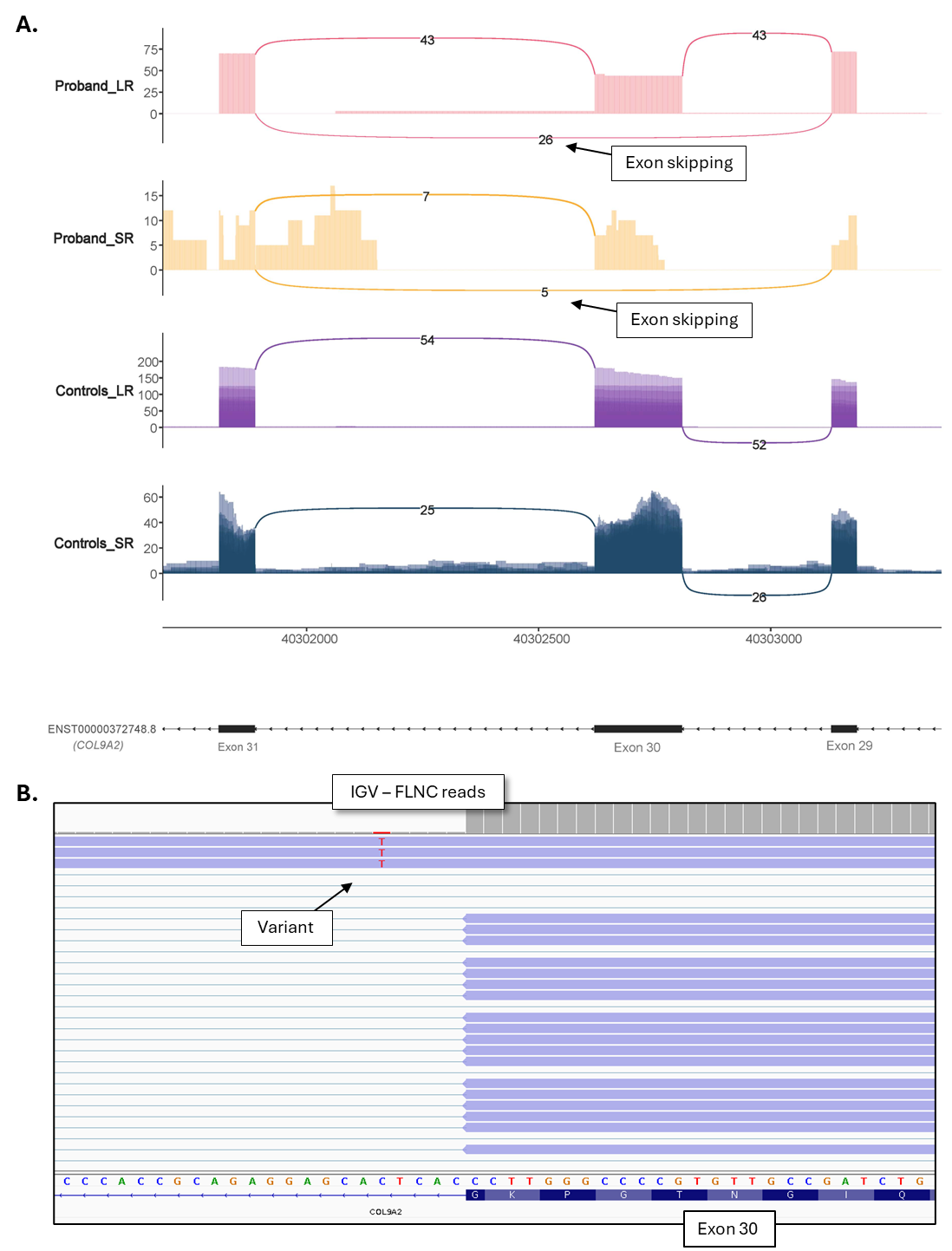
**

**Figure 7.** Transcript visualisation in proband P12. **A.** Sashimi plots comparing splicing patterns between probands and controls. Both short-read (SR) and long-read (LR) data are shown. **B.** Integrated Genomics Viewer (IGV) screenshot of full-length non-chimeric (FLNC) reads from proband. Long read data is presented without clustering.

**P13 - NM_007254.4(*PNKP*):c.1029+2T>C [Heterozygous]:** Short and long-read sequencing detected exon 11 skipping resulting in r.937_1029del, p.(Phe313_Pro343del), absent from controls (**Figure 8A**). Additionally, long-read sequencing identified retention of intron 10 and 11 linked to the variant (**Figure 8B**). While there was some indication of intron retention in the short reads, the intron 10 retention would have been missed as controls also show reads within this intron.

**
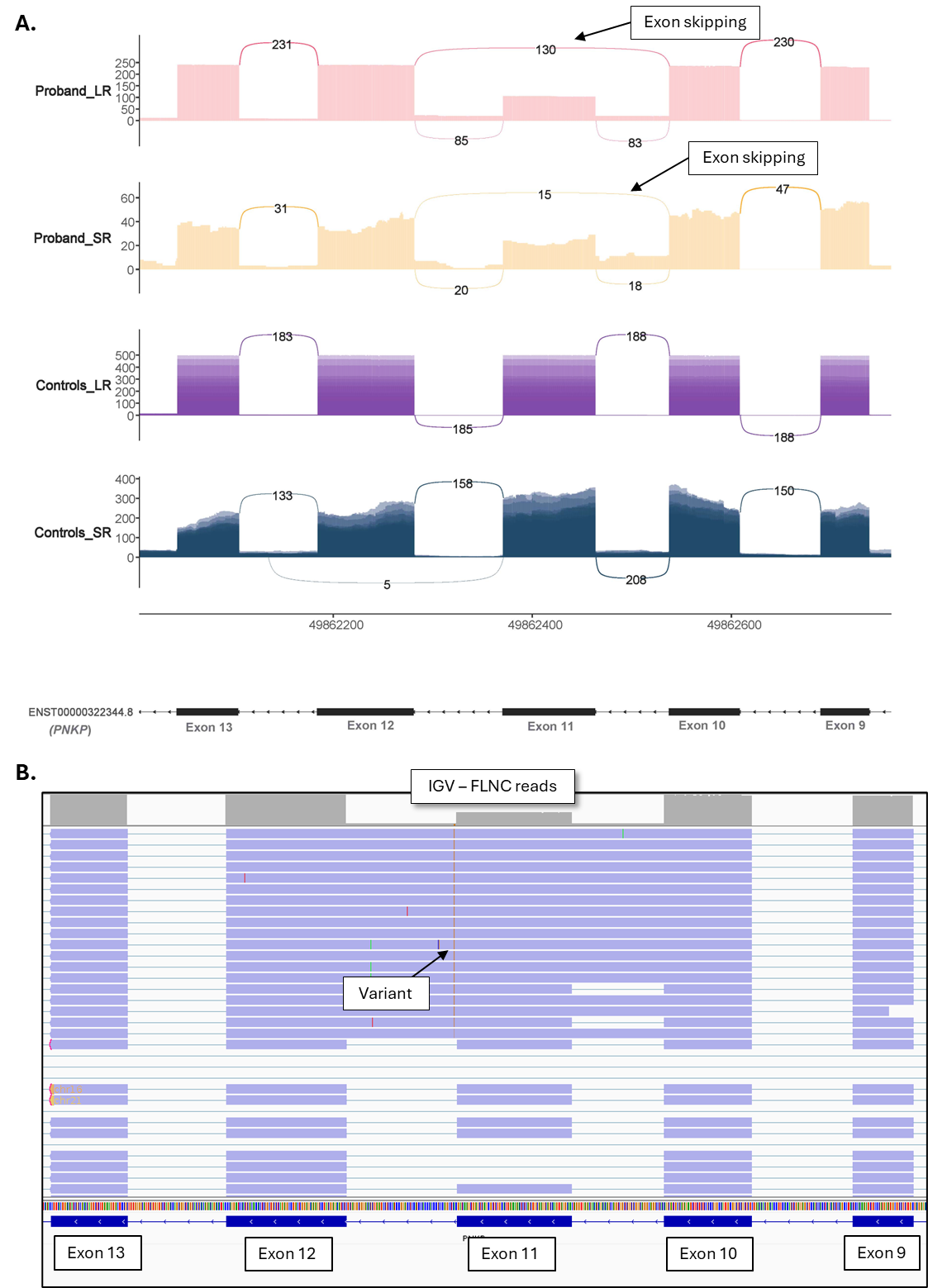
**

**Figure 8.** Transcript visualisation in proband P13. **A.** Sashimi plots comparing splicing patterns between probands and controls. Both short-read (SR) and long-read (LR) data are shown. **B.** Integrated Genomics Viewer (IGV) screenshot of full-length non-chimeric (FLNC) reads from proband. Long read data is presented without clustering.

**P14 - NM_019613.4(*WDR45B*):c.143-5T>A [Homozygous]:** Short and long-read sequencing identified exon 3 skipping, resulting in r.142_244del, p.(Glu48Ter)(**Figure 9A**). Exon 3 skipping is also detected in controls but at very low levels (< 10 reads compared to 100+ in proband).

**
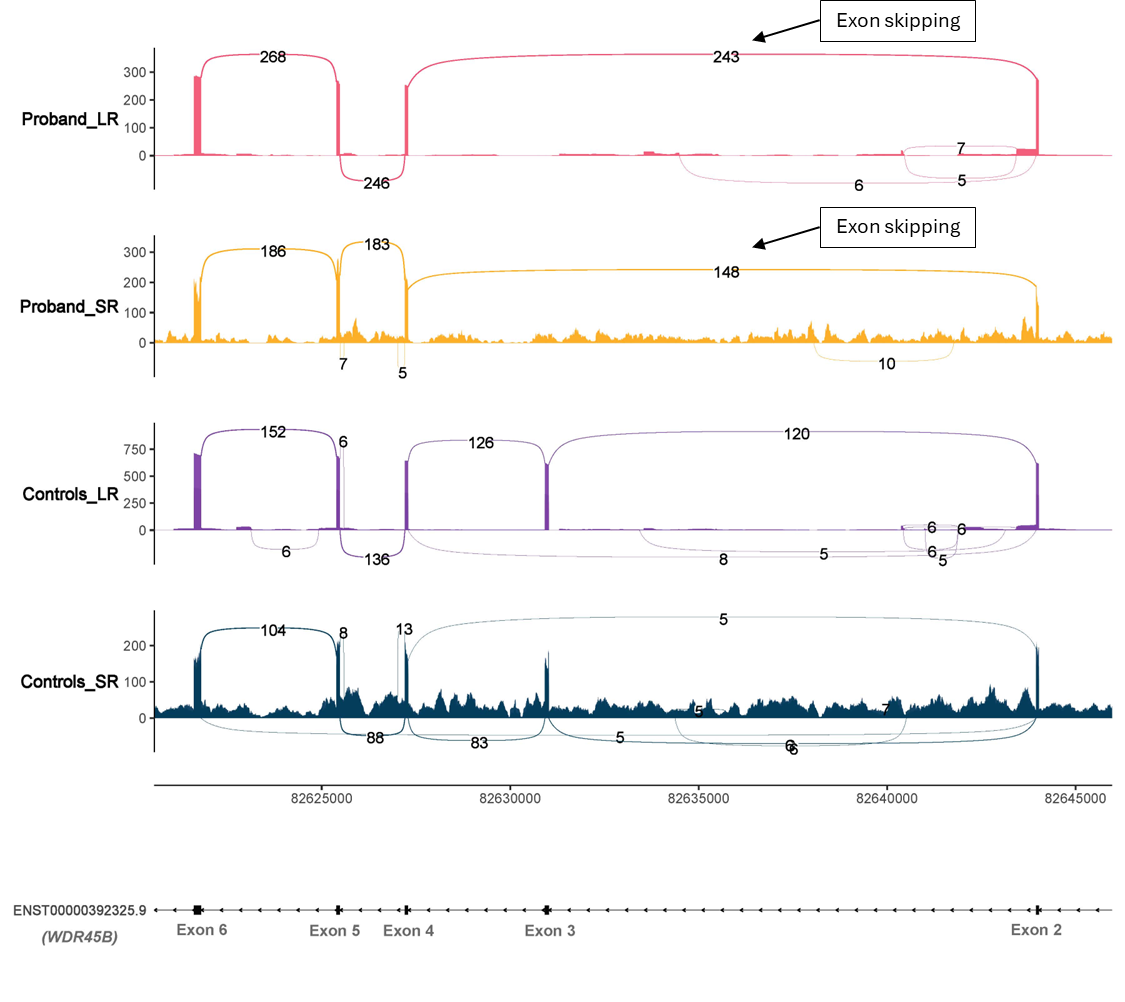
**

**Figure 9.** Sashimi plots comparing splicing patterns between probands and controls. Both short-read (SR) and long-read (LR) data are shown.

**P15 - NM_001378452.1(*ITPR1*):c.1712A>G [Heterozygous]:** Both short and long reads show creation of a new donor site within intron 17 leading to an out-of-frame insertion, r.1713_1714ins1713+1_1713+17, p.(Glu572ValfsTer9). While the long reads did not provide additional information with regards to splicing, it was able to phase the splicing variant with a second variant of interest NM_001378452.1:c.2659C>T (**Figure 10B**).


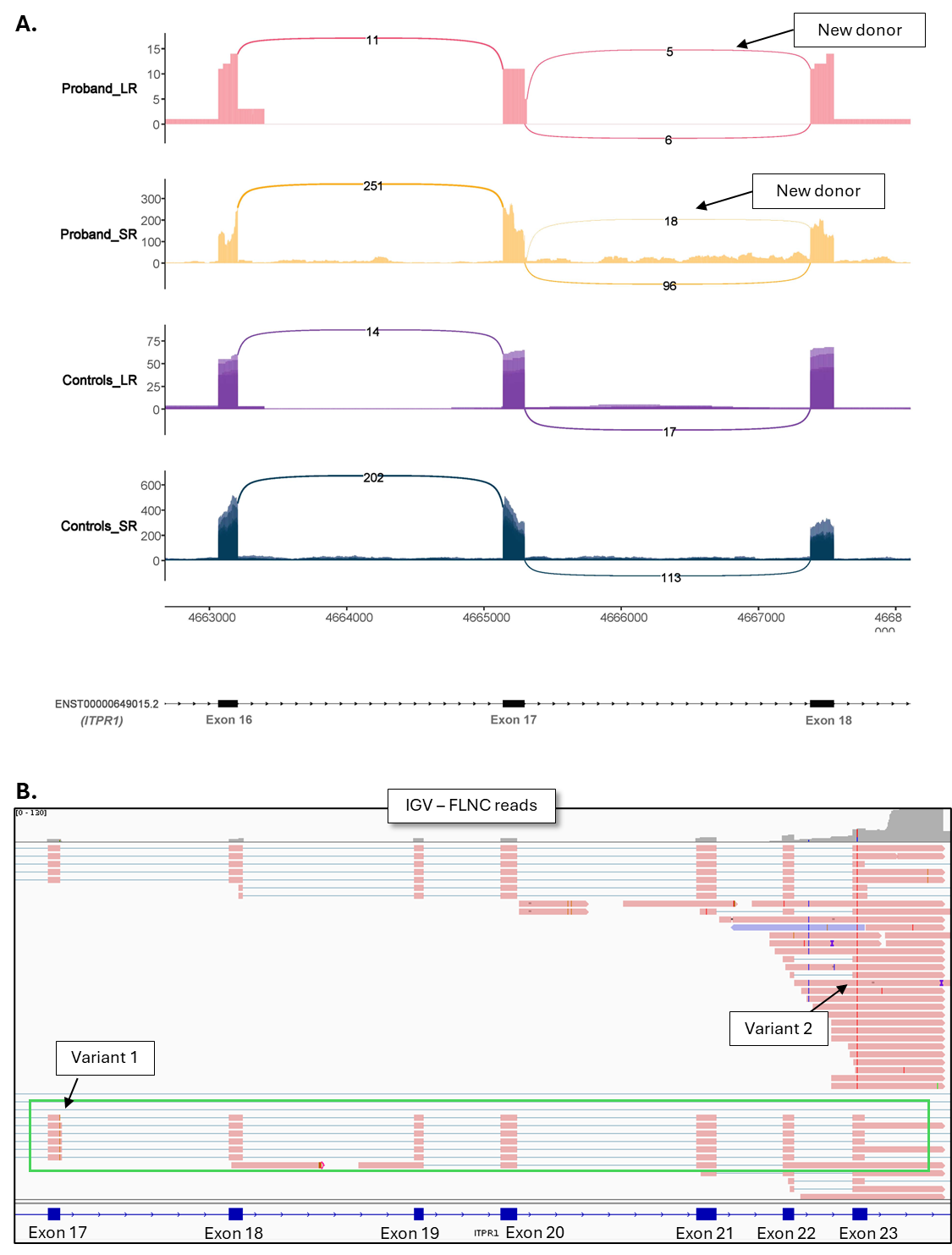


**Figure 10**. Transcript visualisation in proband P15. **A.** Sashimi plots comparing splicing patterns between probands and controls. Both short-read (SR) and long-read (LR) data are shown. **B.** Integrated Genomics Viewer (IGV) screenshot of full-length non-chimeric (FLNC) reads from proband. Variant 1 is splicing variant initially referred for RNA-seq. The green box highlights transcripts containing variant 1, none of which include variant 2, confirming that the two variants are in trans. Long read data is presented without clustering.

**P16 - NM_001145678.3(*KIAA0825*):c.3451_3456+13del [Heterozygous]:** Short-read sequencing identified increased exon 18 skipping in proband. Long-read data lacked sufficient coverage in this sample; however, control samples showed low but detectable coverage of exon 18 and its flanking regions.


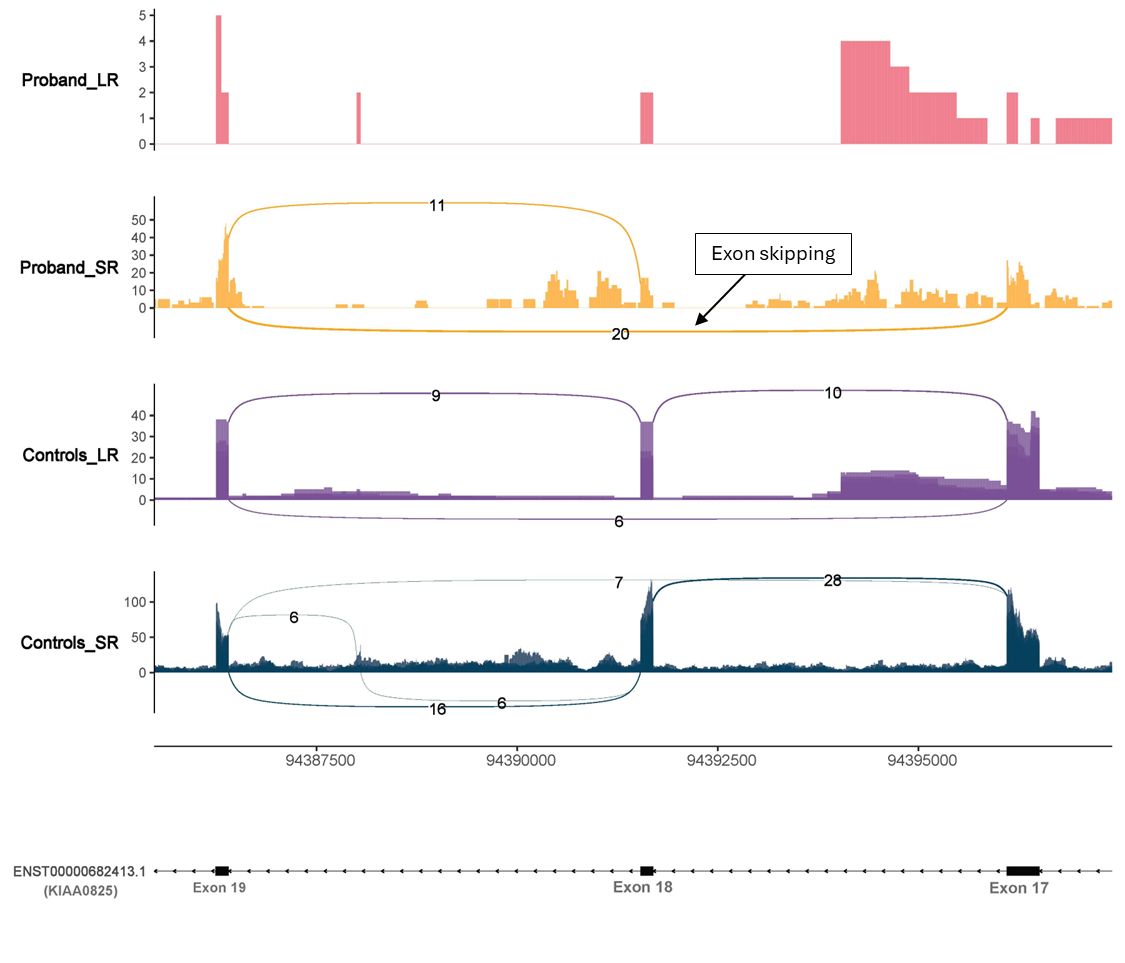


**Figure 11**. Transcript visualisation in proband P16. **A.** Sashimi plots comparing splicing patterns between probands and controls. Both short-read (SR) and long-read (LR) data are shown.

**P17 - NM_004247.4(*EFTUD2*):c.1393A>G, p.(Met465Val) [Heterozygous]:** Both short and long reads show creation of a new donor site within intron 17 leading to an out of frame transcript r.1393_1411del, p.(Ser466AlafsTer3), absent from controls (**Figure 12**). No additional events were detected with LRS.


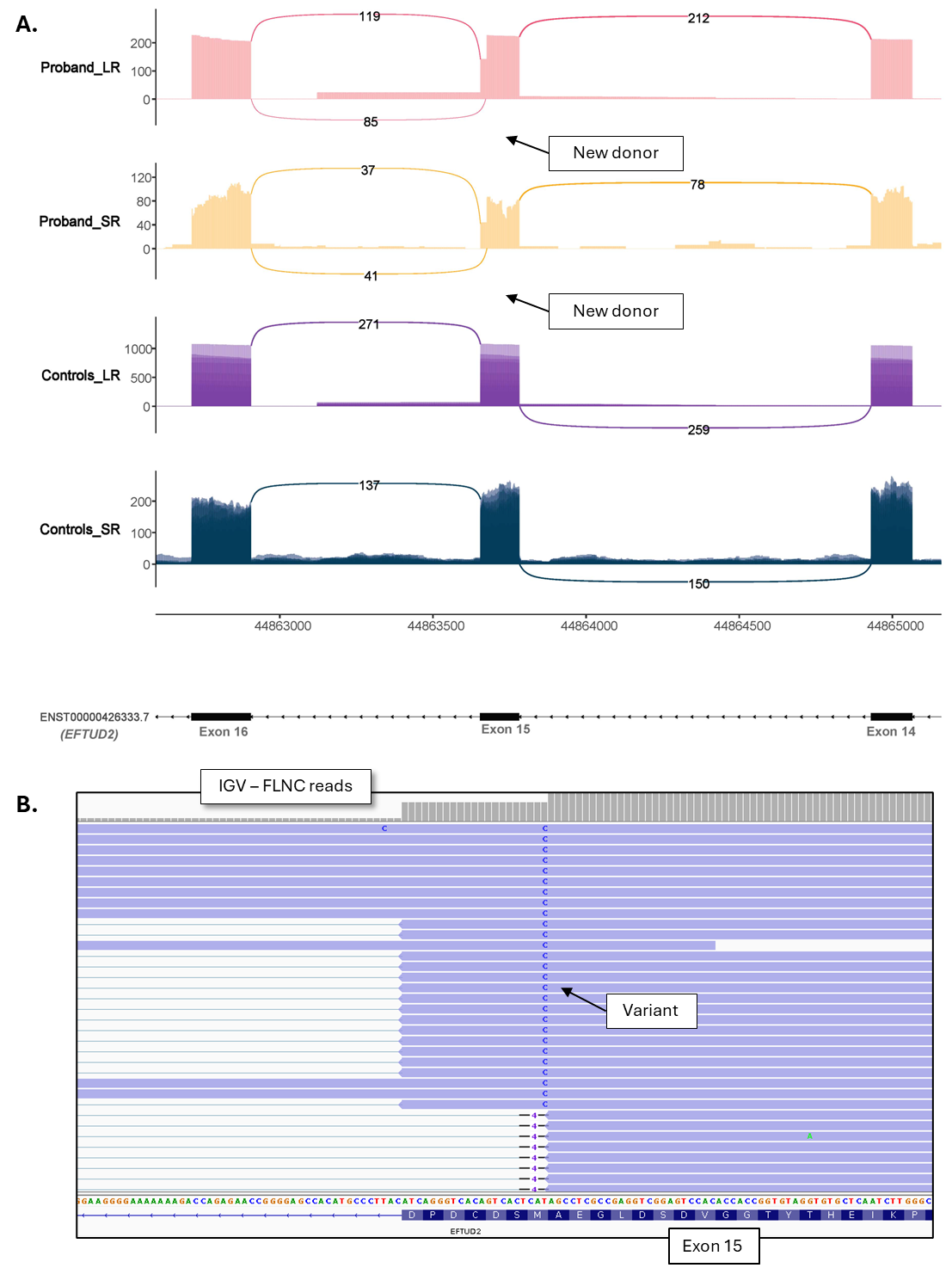


**Figure 12.** Transcript visualisation in proband P17. **A.** Sashimi plots comparing splicing patterns between probands and controls. Both short-read (SR) and long-read (LR) data are shown. **B.** Integrated Genomics Viewer (IGV) screenshot of full-length non-chimeric (FLNC) reads from proband. Long read data is presented without clustering.

**P18 - NM_197968.4(ZMYM2):c.3301+5G>A [Heterozygous]: ]:** Short and long-read sequencing identified exon 20 skipping, resulting in r.3133_3301del, p.(Gly1045LeufsTer4). Long reads also identified exon skipping within other samples at lower levels (**Figure 13**).


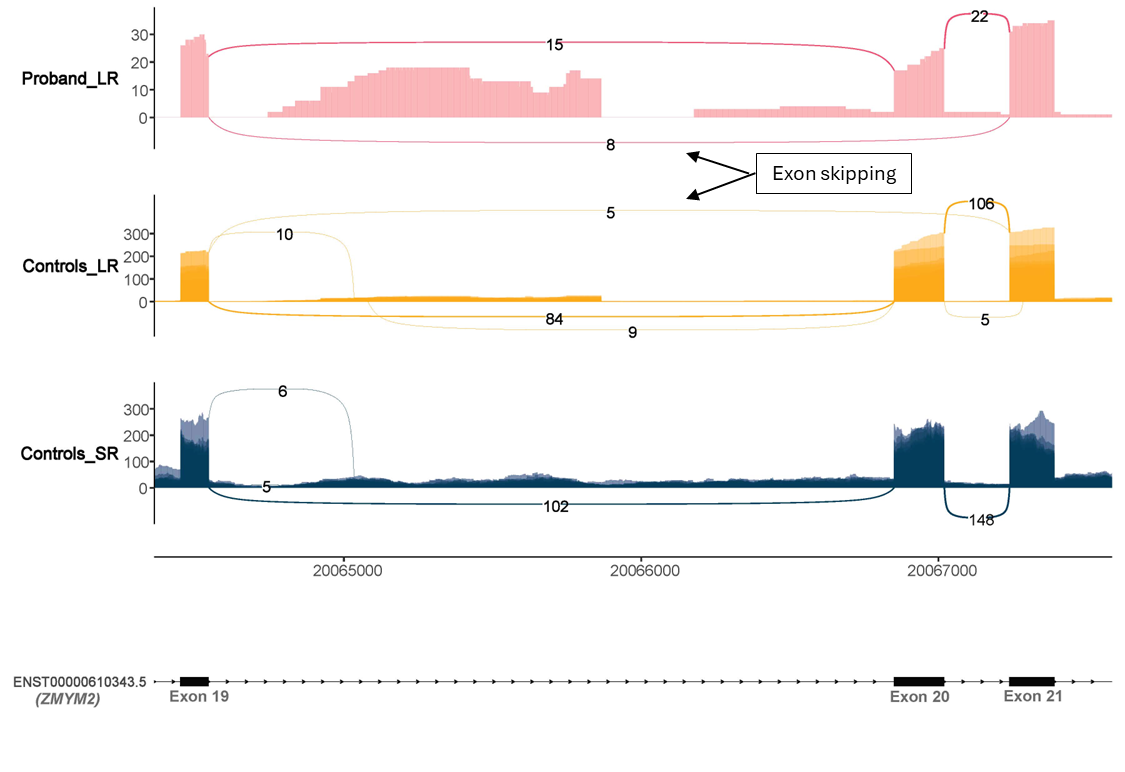


**Figure 13.** Sashimi plots comparing splicing patterns between probands and controls. Both short-read (SR) and long-read (LR) data are shown.

**P19 - NM_001080517.3(*SETD5*):c.-177+1G>A [Heterozygous]:** Both short- and long-read sequencing detect the variant at low levels, but no splicing abnormality is apparent in the sashimi plots (**Figure 14A**). There appears to be donor site loss; however, the intron in question is very large and no LRS reads show full intron retention. Intronic reads are present across all samples, including controls, which complicates interpretation. These factors suggest that while the variant may affect splicing, the event is either too low-level to be confidently detected or may not result in a stable transcript.

**
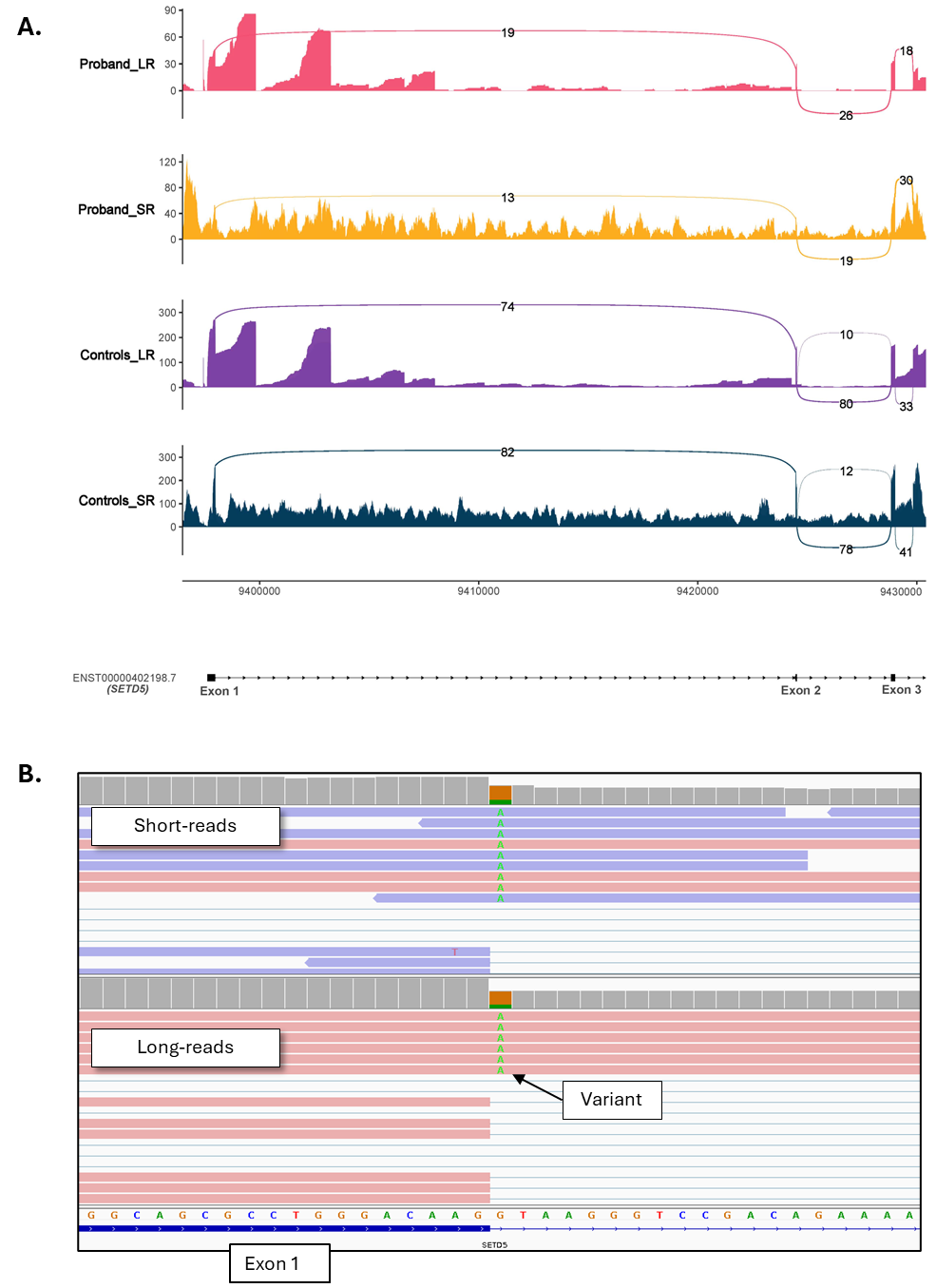
**

**Figure 14.** Transcript visualisation in proband P19. **A.** Sashimi plots comparing splicing patterns between probands and controls. Both short-read (SR) and long-read (LR) data are shown. **B.** Integrated Genomics Viewer (IGV) screenshot of short-read RNAseq and full-length non-chimeric (FLNC) reads from long-read sequencing. Long read data is presented without clustering.

**P20 - NM_000249.4(*MLH1*):c.704A>G, p.(Asp235Gly) [Heterozygous]:** Short and long-read sequencing identified exon 9 skipping, resulting in r.678_790del, p.(Glu227SerfsTer42). Additionally, LRS identified increased exon 9 and 10 skipping at roughly the same ratio of exon 9 skipping. Of note, the variant is also observed in reads with normal junctions, suggesting leaky splicing.

**
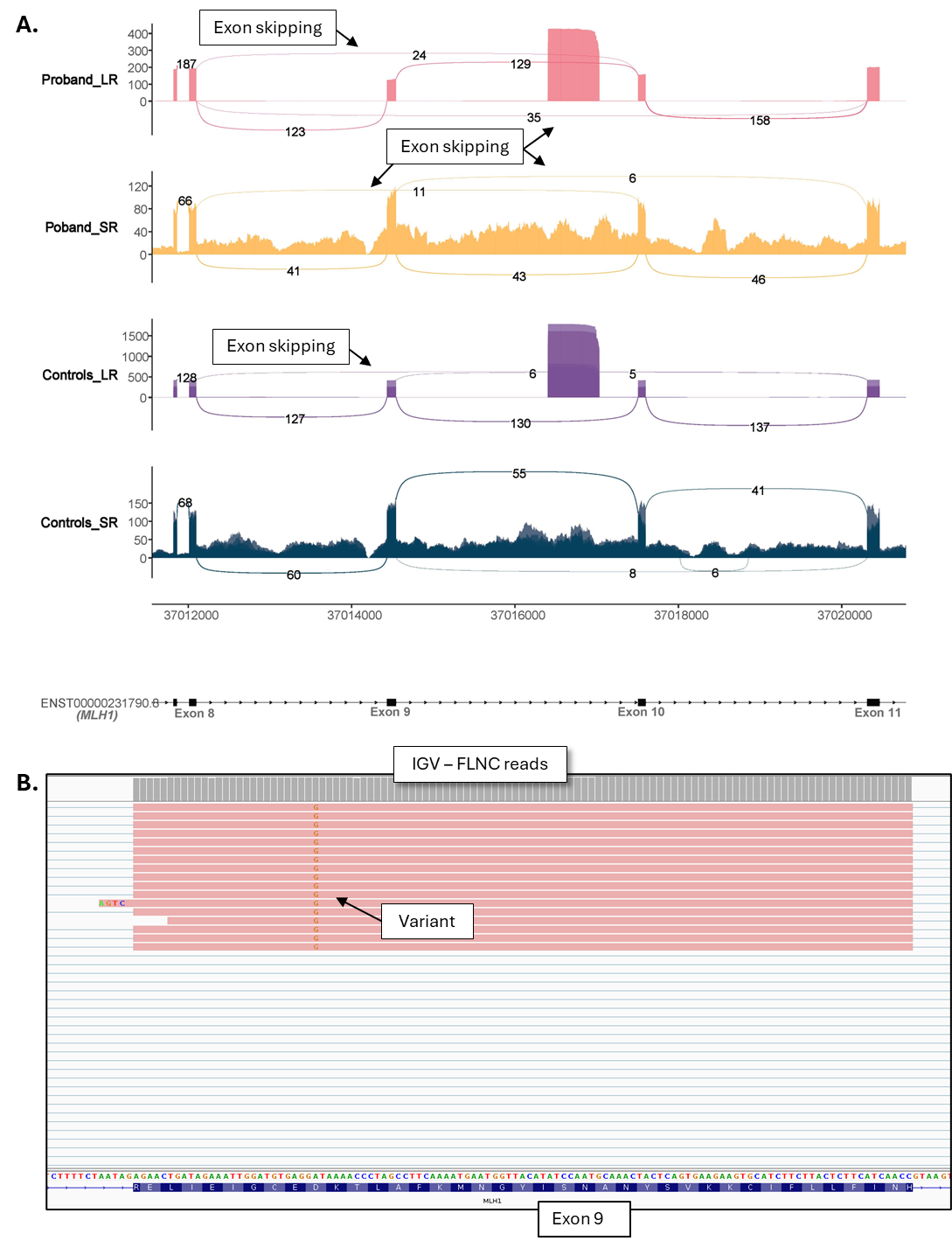
**

**Figure 15.** Transcript visualisation in proband P20. **A.** Sashimi plots comparing splicing patterns between probands and controls. Both short-read (SR) and long-read (LR) data are shown. **B.** Integrated Genomics Viewer (IGV) screenshot of full-length non-chimeric (FLNC) reads from proband. Long read data is presented without clustering.

**P22 - NM_170707(*LMNA*).4:c.1381-5G>A [Heterozygous]:** Short and long-read sequencing identified a new acceptor within intron 7 (**Figure 16A**). This new acceptor was detected at low levels in the SR data with only four supporting reads, whereas 66 supporting reads were observed in the LR data. Interestingly, this event is not visible as a new splice junction in the sashimi plot for the LRS, as the alignment appears to interpret it as an insertion rather than a novel splice site (**Figure 16B**).

**
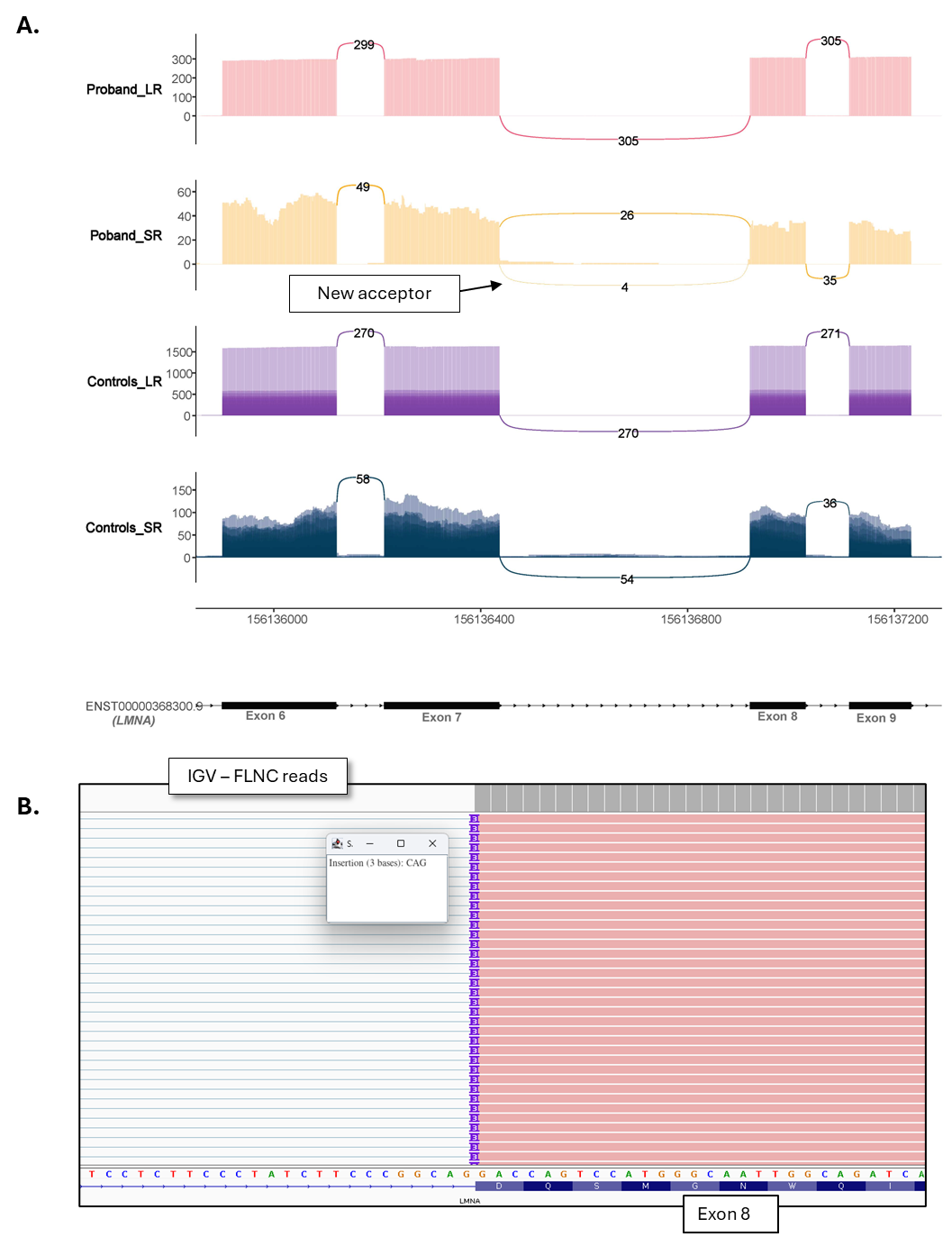
**

**Figure 16.** Transcript visualisation in proband P22. **A.** Sashimi plots comparing splicing patterns between probands and controls. Both short-read (SR) and long-read (LR) data are shown. **B.** Integrated Genomics Viewer (IGV) screenshot of full-length non-chimeric (FLNC) reads from proband. Long read data is presented without clustering.

**P23 - NM_000314.8(PTEN):c.634+3A>C [Heterozygous]:** Long read sequencing identified increased exon 6 skipping which had been missed by the SR RNA-seq. This event is found at low levels in controls (~2%) compared to the proband (~28%). We noted that the short-read RNA-seq failed to detect increased exon 6 skipping due to insufficient coverage across this and downstream exons, for reasons that remain unclear, despite PTEN being highly expressed in this tissue.
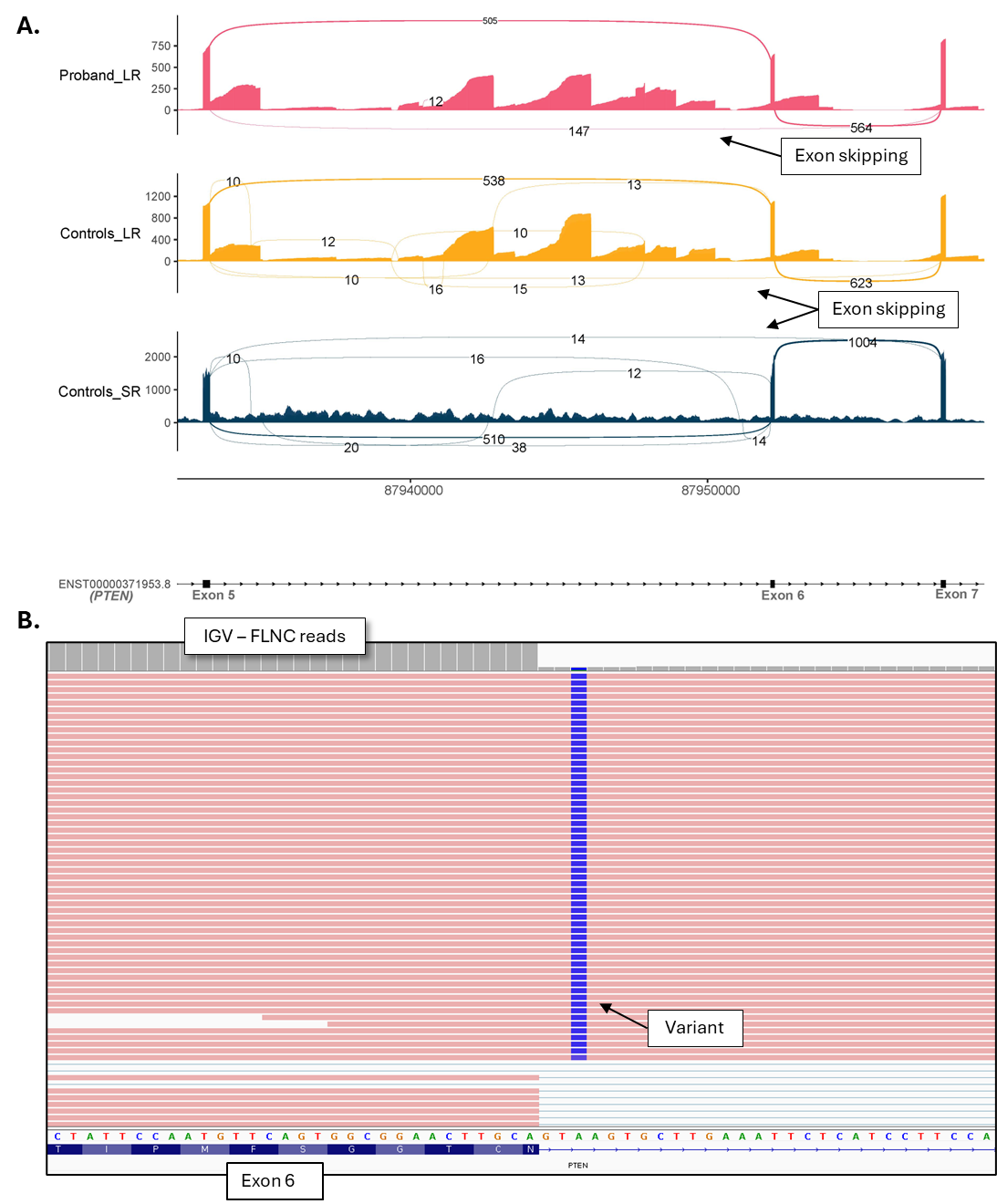
**Figure 17.** Transcript visualisation in proband P23. **A.** Sashimi plots comparing splicing patterns between probands and controls. Both short-read (SR) and long-read (LR) data are shown. **B.** Integrated Genomics Viewer (IGV) screenshot of full-length non-chimeric (FLNC) reads from proband. Long read data is presented without clustering.

**F02 - NM152333.4:c.-120-994_*23708del [Heterozygous]:** Both short and long reads showed *YY1* readthrough transcripts due to the deletion of the last 3 exons of *YY1* and the first two of *SLC25A29* (**Figure 18**). No additional events were detected with LRS.


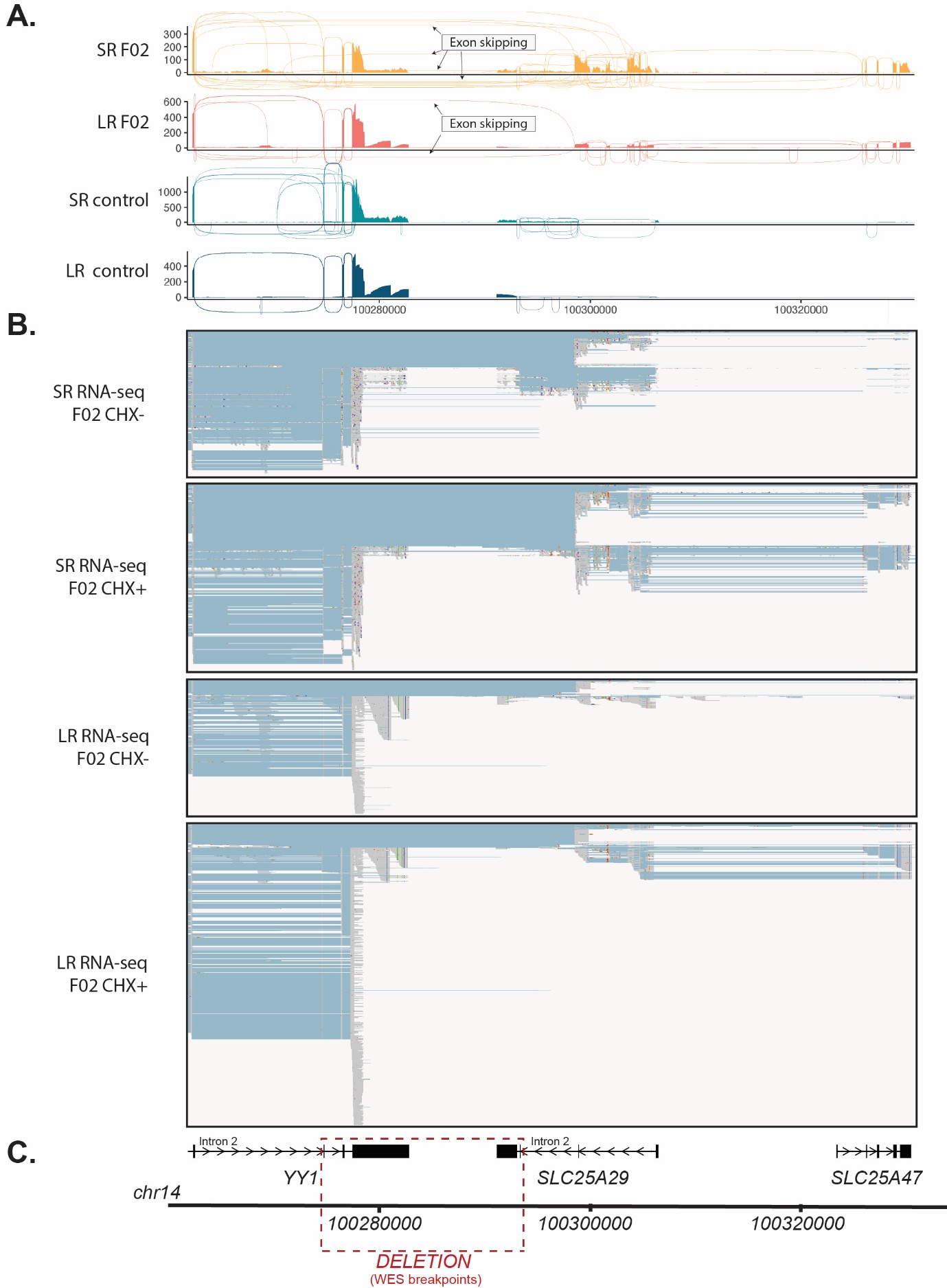


**Figure 18.** Visualization of RNA-seq findings in proband F02. **A.** Sashimi plots comparing splicing patterns between proband fibroblast lines treated with cycloheximide (CHX+) or untreated (CHX-) and controls. **B.** Integrated Genomics Viewer (IGV) screenshot of SR RNA-seq and FLNC reads from proband. **C.** Schematic of genomic coordinates and genes in the locus, and of the expected deletion based on WES data.
